# Emulating the GRADE trial using real world data: retrospective comparative effectiveness study

**DOI:** 10.1136/bmj-2022-070717

**Published:** 2022-10-03

**Authors:** Yihong Deng, Eric C Polley, Joshua D Wallach, Sanket S Dhruva, Jeph Herrin, Kenneth Quinto, Charu Gandotra, William Crown, Peter Noseworthy, Xiaoxi Yao, Timothy D Lyon, Nilay D Shah, Joseph S Ross, Rozalina G McCoy

**Affiliations:** 1 Robert D and Patricia E Kern Center for the Science of Health Care Delivery, Mayo Clinic, Rochester, MN, USA; 2 OptumLabs, Eden Prairie, MN, USA; 3 Department of Public Health Sciences, University of Chicago, Chicago, IL, USA; 4 Department of Environmental Health Sciences, Yale School of Public Health, New Haven, CT, USA; 5 Section of Cardiology, San Francisco Veterans Affairs Health Care System, San Francisco, CA, USA; 6 Department of Medicine, UCSF School of Medicine, San Francisco, CA, USA; 7 Section of Cardiovascular Medicine, Yale School of Medicine, New Haven, CT, USA; 8 Flying Buttress Associates, Charlottesville, VA, USA; 9 Office of Medical Policy, Center for Drug Evaluation and Research, US Food and Drug Administration, Silver Springs, MD, USA; 10 Office of New Drugs, Center for Drug Evaluation and Research, US Food and Drug Administration, Silver Springs, MD, USA; 11 Florence Heller Graduate School, Brandeis University, Waltham, MA, USA; 12 Department of Cardiovascular Medicine, Mayo Clinic, Rochester, MN, USA; 13 Department of Urology, Mayo Clinic, Jacksonville, FL, USA; 14 Delta Air Lines, Atlanta, GA, USA; 15 Department of Internal Medicine, Yale School of Medicine, New Haven, CT, USA; 16 Department of Health Policy and Management, Yale School of Public Health, New Haven, CT, USA; 17 Division of Community Internal Medicine, Geriatrics, and Palliative Care, Department of Medicine, Mayo Clinic, Rochester, MN 55905, USA

## Abstract

**Objective:**

To emulate the GRADE (Glycemia Reduction Approaches in Diabetes: A Comparative Effectiveness Study) trial using real world data before its publication. GRADE directly compared second line glucose lowering drugs for their ability to lower glycated hemoglobin A_1c_ (HbA_1c_).

**Design:**

Observational study.

**Setting:**

OptumLabs® Data Warehouse (OLDW), a nationwide claims database in the US, 25 January 2010 to 30 June 2019.

**Participants:**

Adults with type 2 diabetes and HbA_1c_ 6.8-8.5% while using metformin monotherapy, identified according to the GRADE trial specifications, who also used glimepiride, liraglutide, sitagliptin, or insulin glargine.

**Main outcome measures:**

The primary outcome was time to HbA_1c_ ≥7.0%. Secondary outcomes were time to HbA_1c_ >7.5%, incident microvascular complications, incident macrovascular complications, adverse events, all cause hospital admissions, and all cause mortality. Propensity scores were estimated using the gradient boosting machine method, and inverse propensity score weighting was used to emulate randomization of the treatment groups, which were then compared using Cox proportional hazards regression.

**Results:**

8252 people were identified (19.7% of adults starting the study drugs in OLDW) who met eligibility criteria for the GRADE trial (glimepiride arm=4318, liraglutide arm=690, sitagliptin arm=2993, glargine arm=251). The glargine arm was excluded from analyses owing to small sample size. Median times to HbA_1c_ ≥7.0% were 442 days (95% confidence interval 394 to 480 days) for glimepiride, 764 (741 to not calculable) days for liraglutide, and 427 (380 to 483) days for sitagliptin. Liraglutide was associated with lower risk of reaching HbA_1c_ ≥7.0% compared with glimepiride (hazard ratio 0.57, 95% confidence interval 0.43 to 0.75) and sitagliptin (0.55, 0.41 to 0.73). Results were consistent for the secondary outcome of time to HbA_1c_ >7.5%. No significant differences were observed among treatment groups for the remaining secondary outcomes.

**Conclusions:**

In this emulation of the GRADE trial, liraglutide was statistically significantly more effective at maintaining glycemic control than glimepiride or sitagliptin when added to metformin monotherapy. Generating timely evidence on medical treatments using real world data as a complement to prospective trials is of value.

## Introduction

Type 2 diabetes is a common serious chronic health condition, impacting 11.3% (37.3 million) of the US population^1^ and 9.3% (463 million) of people worldwide.^2^ Moderate glycemic control, defined by achieving glycated hemoglobin (HbA_1c_) between 7% and 8%, improves microvascular and macrovascular outcomes.^3^ ^4^ Current clinical practice guidelines recommend targeting HbA_1c_ <7% for most non-pregnant adults.^5^ Timely and appropriate treatment intensification is fundamental to maintaining glycemic control^6^ and preventing complications.^7^ ^8^ ^9^ ^10^ Metformin is the preferred glucose lowering drug owing to its efficacy, tolerability, and low cost.^11^ ^12^ ^13^ ^14^ Type 2 diabetes is, however, a progressive disease, and most patients ultimately require intensification of treatment. Recent US population level estimates suggest that nearly one third of people with HbA_1c_ ≥7% are treated with only one glucose lowering drug^15^ and as such would benefit from treatment intensification. Clinical practice guidelines advise that choice of second line treatment should be informed by clinical and situational considerations specific to each individual, recognizing the knowledge gaps stemming from the lack of direct comparisons of currently available second line drugs.^11^ ^12^ ^13^ ^14^

The GRADE (Glycemia Reduction Approaches in Diabetes: A Comparative Effectiveness Study) trial is a recently completed, but still unpublished, pragmatic, randomized, parallel arm clinical trial that seeks to address this knowledge gap by comparing four second line glucose lowering drugs among adults with moderately uncontrolled type 2 diabetes who are in receipt of metformin monotherapy.^16^ ^17^ The drugs represent four classes: glimepiride (sulfonylurea), sitagliptin (dipeptidyl-peptidase 4 inhibitor), liraglutide (glucagon-like peptide-1 receptor agonist), and insulin glargine (basal analog insulin). The GRADE trial was designed (2008) and launched (July 2013) before US Food and Drug Administration approval of sodium-glucose cotransporter-2 inhibitors and several cardiovascular outcomes trials that showed reduction in atherosclerotic cardiovascular and kidney disease outcomes with use of glucagon-like peptide-1 receptor agonists, and in heart failure and kidney disease outcomes with use of sodium-glucose cotransporter-2 inhibitors. This highlights a key limitation of large prospective randomized controlled trials: such trials are time consuming to conduct, potentially hindering the ability to answer questions in a clinically meaningful time frame. Thus, it is of value to efficiently generate timely evidence on medical treatments using observational research methods applied to real world data as a complement to prospective trials.

Advances in the quantity, quality, and granularity of real world data, combined with improvements in statistical methods used to account for confounding, treatment allocation bias, and time related bias, have provided opportunities to use large scale real world data to inform the understanding of drug effectiveness and safety. Ideally, studies using real world data would be conducted before the publication of the results from randomized controlled trials, thereby minimizing potential biases that could be introduced by trying to replicate known results from such trials. As an illustrative test case of the opportunities and limitations of using observational research methods to emulate randomized controlled trials, and building on parallel analyses emulating the PRONOUNCE (A Trial Comparing Cardiovascular Safety of Degarelix Versus Leuprolide in Patients With Advanced Prostate Cancer and Cardiovascular Disease) trial,^18^ we used claims and laboratory results data from OptumLabs® Data Warehouse (OLDW), a deidentified national dataset of privately insured and Medicare Advantage beneficiaries, to emulate the GRADE trial. We used published^16^ ^19^ and publicly available^17^ information on the GRADE trial’s study design to emulate the methods and anticipated results as closely as possible, with the goal of directly comparing the effectiveness of glimepiride, sitagliptin, liraglutide, and insulin glargine in achieving and maintaining HbA_1c_ <7.0% among adults with type 2 diabetes and HbA_1c_ 6.8-8.5% while in receipt of metformin monotherapy. We also examined the secondary metabolic, microvascular, macrovascular, and safety endpoints planned in the GRADE trial as feasible using the data available within OLDW. Our study therefore had two complementary objectives. First, a clinical objective, to examine four second line glucose lowering drugs for lowering or maintaining, or both, HbA_1c_ <7.0%, filling an important clinical knowledge gap in the comparative effectiveness of these commonly used and guideline recommended drug classes. Second, a methodologic objective, to ascertain whether routinely available claims data can be used to emulate a prospective randomized clinical trial ahead of its publication, filling important methodologic and regulatory policy needs in the use of real world data to predict clinical trial results.

## Methods

### Study design

We retrospectively analyzed medical and pharmacy claims data from OLDW, a deidentified claims dataset that includes healthcare utilization information for beneficiaries of private health plans (adults of working age and their dependents) and Medicare Advantage plans. The latter are Medicare approved plans offered by private companies to beneficiaries who are eligible for Medicare (eg, adults aged ≥65 years, individuals with disability, people with end stage kidney disease) as a private alternative to Original Medicare. Just as with private insurance, Medicare Advantage plans typically bundle medical and pharmacy coverage. OLDW contains longitudinal health information on enrollees in these health plans, representing a diverse mixture of ages, ethnicities, and geographic regions across the US.^20^ ^21^ The study is reported according to the Reporting of studies Conducted using Observational Routinely-collected Data (RECORD) reporting guideline.^22^

### Study population

We first assembled a cohort of adults (≥18 years) who initially started glimepiride, sitagliptin, liraglutide, or insulin glargine between 25 January 2010 (date of liraglutide approval by the FDA; remaining study drugs were approved earlier) and 30 June 2019 (see supplemental figure S1). The index date was set to the date of the first claim for the study drug. People who started ≥2 study drugs on the index date were excluded. Individuals were required to be adherent to metformin for ≥8 weeks before that first study drug fill date. This was established by identifying all metformin fills before the index date, establishing continuous treatment episodes based on prescription fill dates and the days’ supply for each fill (allowing up to 30 day gap between fills), and requiring that the last metformin treatment episode before the index date be at least eight weeks. To ensure consistent and adequate capture of baseline comorbidities and treatment data, people were required to have six months of continuous enrollment with medical and pharmacy coverage before the index date. We excluded those with fills for any glucose lowering drugs other than metformin during the baseline period and those with type 1 diabetes, defined using ICD-9 and ICD-10 (international classification of diseases, ninth and 10th revisions, respectively) codes. Individuals were further required to have valid personal (age, sex, region) data and HbA_1c_ results both within three months before the index date (baseline HbA_1c_) and during follow-up. Next, we adapted the eligibility criteria for the GRADE trial^16^ ^17^ ^19^ and applied these to beneficiaries included in OLDW, as detailed in supplemental table S1. Supplemental tables S2 and S3 summarize the relevant diagnosis codes and drugs. All eligible individuals in OLDW were included in the cohort.

### Outcomes

The primary outcome was time to primary metabolic failure, calculated as days to HbA_1c_ ≥7.0% while treated with the assigned drug, with the period of eligibility starting at month 3 after the index date (analogous to the first quarterly HbA_1c_ assessment in the GRADE trial). Unlike the GRADE trial protocol, we did not require a confirmatory HbA_1c_ owing to variation in real world HbA_1c_ testing intervals. To assess for potential bias in outcome ascertainment as the result of different frequencies of HbA_1c_ testing and varying intervals between tests among the treatment groups, we compared the number, frequency, and timing of available HbA_1c_ test results and found no difference between the groups (see supplemental table S4). Because testing frequency is guided by baseline HbA_1c_, we also examined intervals between sequential HbA_1c_ tests stratified by baseline HbA_1c_ and found no differences between the treatment groups (see supplemental table S5).

Secondary metabolic, cardiovascular, and microvascular outcomes were analyzed as specified in the GRADE trial’s statistical analysis plan^17^ if they were feasible to ascertain using claims data (see supplemental table S6). Individuals were followed until they experienced the outcome of interest, anticipated follow-up duration of the trial (seven years), end of the study period (31 July 2019), end of insurance coverage, or death. Individuals with outcomes observed while being treated with the assigned regimen, were followed until they discontinued the assigned drug (defined as not refilling a drug after 30 days of the end of last treatment episode), with the goal of emulating the definitions of these outcomes in the GRADE trial (ie, while being treated with the originally assigned drugs).^16^

### Independent variables

Patient individual level age, sex, race or ethnicity, and annual household income were identified from OLDW enrollment files at the time of the index date. Detailed description of the source data for these variables is available in the supplemental methods. Comorbidities (ascertained from all claims during six months preceding the index date) included retinopathy, nephropathy, neuropathy, coronary artery disease, cerebrovascular disease, peripheral vascular disease, heart failure, and previous severe hypoglycemia and hyperglycemia, as detailed in supplemental table S2. Specialties of treating physicians were categorized as primary care, endocrinology, cardiology, nephrology, other, and unknown. Baseline drugs, included as surrogates for burden of complications, were identified from fills in the six months preceding the index date (see supplemental table S3).

### Statistical analysis

Inverse probability of treatment weighting was used to balance the differences in baseline characteristics among the treatment groups. Propensity scores were used as probability of treatment; these propensity score weights were estimated using generalized boosted models including the baseline variables presented in table 1. Using generalized boosted models involves an iterative process with multiple regression trees to capture complex and non-linear relations between treatment assignments and the pretreatment covariates, with the propensity score model leading to the best balance among the treatment groups.^23^ The supplemental methods provide additional detail on the models. We calculated stabilized weights with multiple treatments by dividing the marginal probability of treatment by the propensity score of treatment received.^24^ Supplemental figure S2 shows the distribution of weights. Standardized mean differences were used to assess the balance of covariates after weighting; a standardized mean difference ≤0.1 was considered a good balance and ≤0.2 was considered acceptable.^25^ Before evaluation of the outcomes, we examined the weighted sample sizes and ability to account for baseline confounding to determine the feasibility of including each treatment group.

**Table 1:** Baseline characteristics in weighted cohort. Values are numbers (percentages) unless stated otherwise

Characteristics	Glimepiride	Liraglutide	Sitagliptin	Largest SMD
Weighted No	4168	572	2800	
Mean (SD) age (years)	62.0 (11.1)	60.5 (10.4)	62.0 (11.0)	0.14
Sex:				
Women	2009 (48.2)	289 (50.5)	1374 (49.1)	0.05
Men	2159 (51.8)	283 (49.5)	1427 (50.9)	
Race or ethnicity:				
White	2695 (64.7)	376 (65.8)	1798 (64.2)	0.09
Black	536 (12.9)	79 (13.7)	355 (12.7)	
Hispanic	513 (12.3)	72(12.6)	353 (12.6)	
Asian	266 (6.4)	28 (4.9)	185 (6.6)	
Unknown	159 (3.8)	17 (3.0)	108 (3.9)	
Annual household income ($):				
<40 000	989 (23.7)	123 (21.6)	647 (23.1)	0.10
40 000-74 999	1145 (27.5)	175 (30.7)	766 (27.3)	
75 000-124 999	1153 (27.7)	164 (28.6)	772 (27.6)	
125 000-199 999	485 (11.6)	63 (11.0)	336 (12.0)	
≥200 000	185 (4.4)	26 (4.5)	139 (4.9)	
Unknown or missing	211 (5.1)	21 (3.7)	142 (5.1)	
Mean (SD) baseline HbA_1c_	7.6 (0.5)	7.6 (0.5)	7.6 (0.5)	0.06
Baseline HbA_1c_ categories (%):				
6.8-6.9	336 (8.1)	59 (10.3)	233 (8.3)	0.09
7.0-7.9	2630 (63.1)	364 (63.7)	1786 (63.8)	
8.0-8.5	1202 (28.8)	149 (26.0)	782 (27.9)	
Mean (SD) baseline creatinine (mg/dL)*	0.9 (0.2)	0.9 (0.2)	0.9 (0.2)	0.02
Baseline comorbidities:				
Nephropathy	363 (8.7)	55 (9.5)	229 (8.2)	0.05
Retinopathy	196 (4.7)	31 (5.4)	135 (4.8)	0.03
Neuropathy	505 (12.1)	75 (13.2)	326 (11.7)	0.05
Hyperglycemia	2 (0.0)	0	0	0.03
Hypoglycemia	2 (0.0)	0	0	0.03
Coronary artery disease	355 (8.5)	53 (9.2)	224 (8.0)	0.04
Chronic kidney disease	121(2.9)	18 (3.1)	82 (2.9)	0.01
Cerebrovascular disease	117 (2.8)	16 (2.8)	80 (2.8)	0.00
Peripheral vascular disease	190 (4.6)	28 (4.9)	130 (4.6)	0.02
Baseline drugs:				
ACE inhibitor	1819 (43.6)	250 (43.7)	1206 (43.1)	0.01
ARB	1110 (26.6)	157 (27.4)	755 (27.0)	0.02
ACE inhibitor or ARB	2850 (68.4)	400 (69.9)	1906 (68.0)	0.04
Direct oral anticoagulant	57 (1.4)	9 (1.5)	40 (1.4)	0.01
Statin	2853 (68.5)	374 (65.4)	1937 (69.2)	0.08
Non-statin lipid lowering drugs	549 (13.2)	85 (14.9)	382 (13.6)	0.05
Warfarin	83 (2.0)	12 (2.0)	48 (1.7)	0.02
Peripheral neuropathy drugs	410 (9.8)	74 (12.9)	281 (10.0)	0.09
Specialty of treating physicians:				
Primary care	3202 (76.8)	424 (74.1)	2145 (76.6)	0.10
Endocrinology	174 (4.2)	27 (4.7)	120 (4.3)	
Cardiology	28 (0.7)	2 (0.4)	19 (0.7)	
Nephrology	6 (0.1)	0	5 (0.2)	
Other	274 (6.6)	45 (7.9)	179 (6.4)	
Unknown	483 (11.6)	74 (12.9)	333 (11.9)	
Year of cohort entry:				
2010	237 (5.7)	29 (5.0)	167 (6.0)	0.13
2011	241 (5.8)	25 (4.4)	168 (6.0)	
2012	297 (7.1)	41 (7.2)	204 (7.3)	
2013	388 (9.3)	65 (11.3)	268 (9.6)	
2014	464 (11.1)	65 (11.4)	318 (11.4)	
2015	449 (10.8)	58 (10.1)	293 (10.4)	
2016	575 (13.8)	75 (13.0)	380 (13.6)	
2017	761 (18.3)	97 (16.9)	497 (17.8)	
2018	632 (15.2)	91 (15.9)	422 (15.1)	
2019	126 (3.0)	27 (4.7)	84 (3.0)	

$ 1.00 (£0.87; €1.01).

ARB=angiotensin II receptor blockers; ACE=angiotensin converting enzyme; HbA_1c_=hemoglobin A_1c_; SD=standard deviation; SMD=standardized mean difference.

* Creatine was not included in the models as only a subset of the population had available data (reported in the table). 1 mg/dL×88.4=1 μmol/L.

The cumulative incidence of the primary (time to first HbA_1c_ ≥7.0) and secondary (time to first HbA_1c_ >7.5%) metabolic failures within each treatment arm was estimated with the inverse probability of treatment weighting Kaplan-Meier method. We used the propensity score weighted Cox proportional hazards regression models adjusted by baseline HbA_1c_ values to compare the outcomes between treatment groups. As the primary outcome can be only observed from the third month, we set the at risk time for the proportional hazards model as three months after the index date. Results are presented as median times to metabolic failure and expected proportions of people experiencing metabolic failure at one and two years. All pairwise comparisons between the treatment groups were estimated, and we applied the Holm method to adjust the P values for multiple testing. We tested the proportional hazards assumption using Schoenfeld residuals. Similar analyses were performed for other time-to-event outcomes. The at risk start time for modeling secondary metabolic, cardiovascular, and microvascular disease outcomes was set at the study index date. Repeated measures HbA_1c_ trends by treatment group were estimated by using the inverse probability of treatment weighting mean HbA_1c_ results by treatment group in three month time intervals. The follow-up time by treatment arm was estimated using the same propensity score weights as the primary analysis and the inverse probability of treatment weighting Kaplan-Meier method for the censoring distribution.^26^

All primary analyses were conducted using the per protocol censoring approach for the primary outcome and for the secondary outcomes of secondary metabolic failure and insulin initiation, censoring at the time of treatment drug discontinuation, disenrollment from the health plan, end of study period, or death, whichever came first (see supplemental figure S3). Time receiving treatment for each drug was determined by calculating continuous coverage episodes based on available fills—the same as for baseline metformin treatment. Remaining secondary outcomes were analyzed using the intention-to-treat censoring approach, censoring the participant at the time of health plan disenrollment, end of study, or death, which ever came first. P<0.05 was considered statistically significant for all two sided tests. All analyses were performed using SAS 9.4 (SAS Institute, Cary, NC) and R version 4.0.2.(R Foundation).

### Subgroup analyses

A priori defined subgroup analyses were performed as a function of baseline HbA_1c_ (<7.0% *v* ≥7.0%), age group (<65 years, ≥65 years), sex (men *v* women), and race or ethnicity (white, black, Hispanic, Asian).

### Sensitivity analyses

First, to examine the comparative effectiveness of study drugs while treated only with them and not with any other drug, accounting for real world treatment practices, we repeated all analyses using the as treated censoring approach, censoring at the time a new drug class was added, the assigned drug was discontinued, health plan disenrollment, end of study, or death, which ever came first (see supplemental figure S3). Second, we assessed residual confounding by testing a falsification endpoint that was unlikely to be associated with the studied drugs: diagnosis of pneumonia (see supplemental table S2) during the follow-up period.

### Patient and public involvement

Patients were not involved in the design, conduct, or dissemination of this study. However, this study was informed by the need to identify preferred glucose lowering treatment strategies in the absence of direct comparisons across the examined drugs; and to examine whether and how data collected in the process of routine patient care can be used to emulate prospective clinical trials. Because this study seeks to inform drug regulatory policy and procedures, investigators from the FDA contributed to the design of the study and interpretation of study findings; they are included as coauthors on this publication.

## Results

### Study population

We identified 18 365 adults with type 2 diabetes who started glimepiride, 12 818 who started sitagliptin, 5021 who started liraglutide, and 5659 who started insulin glargine and had the required baseline enrollment and available HbA_1c_ results (see supplemental figure S1). Eligibility criteria of the GRADE trial were met by 19.7% (8252 of 41 863) of these individuals, ranging from 4.4% (251 of 5659) using glargine to 23.5% (4318 of 18 365) using glimepiride. The most prevalent reasons for ineligibility (see supplemental table S7) were HbA_1c_ outside the prespecified range (ranging from 51.7% (6631 of 12 818) of individuals using sitagliptin to 81.1% (4591 of 5659) using glargine) and not being treated with metformin monotherapy at the time of study drug initiation (ranging from 43.5% (7997 of 18 365) of individuals using glimepiride to 68.5% (3874 of 5659) using glargine). The final cohort comprised 4318 individuals in the glimepiride arm, 2993 in the sitagliptin arm, 690 in the liraglutide arm, and 251 in the glargine arm (see supplemental table S8 for all included drugs).

Supplemental table S9 shows baseline characteristics of the included individuals before weighting. Across the four treatment groups, there were significant differences (largest standardized mean difference >0.2) in age, race or ethnicity, annual household income, and prescribing physician specialty. Individuals in the liraglutide arm were more likely to be younger, white, on a higher income, and treated by an endocrinologist than those in the other treatment arms. Individuals in the glargine arm were most likely to be on a low income and they had the highest prevalence of all examined comorbidities.

The glargine arm was excluded from all analyses because of small sample size (n=251, weighted n=179) and inability to achieve good control of confounders after weighting. The propensity score model was estimated on the remaining three groups. After weighting, mean participant ages were 62.0 years (standard deviation (SD) 11.1 years) in the glimepiride arm, 62.0 (SD 11.0) years in the sitagliptin arm, and 60.5 (SD 10.4) years in the liraglutide arm (table 1). Women comprised 48.2% (2009 of 4168) of the glimepiride arm, 49.1% (1374 of 2800) of the sitagliptin arm, and 50.5% (289 or 572) of the liraglutide arms. White people comprised 64.7% (2695 of 4168), 64.2% (1798 of 2800), and 65.8% (376 of 572) of the treatment arms, respectively. Mean baseline HbA_1c_ levels were 7.63% (SD 0.48%), 7.61% (SD 0.47%), and 7.60% (SD 0.48%), respectively. Supplemental table S10 presents the pairwise standardized mean differences for all baseline covariates; all values were <0.2.

### Primary metabolic failure (HbA_1c_ ≥7.0%)

**Fig 1:**
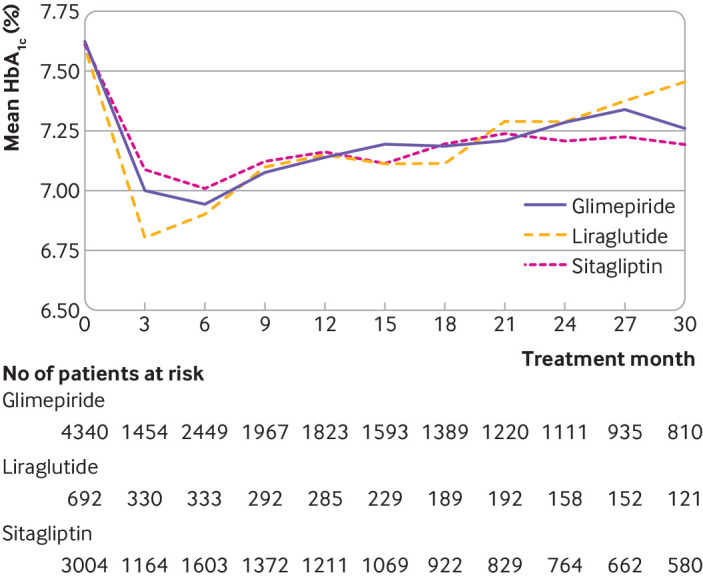
Mean hemoglobin A_1c_ (HbA_1c_) levels over time. Results are based on observed receipt of treatment trajectories, with no imputation of missing HbA_1c_ levels

**Fig 2:**
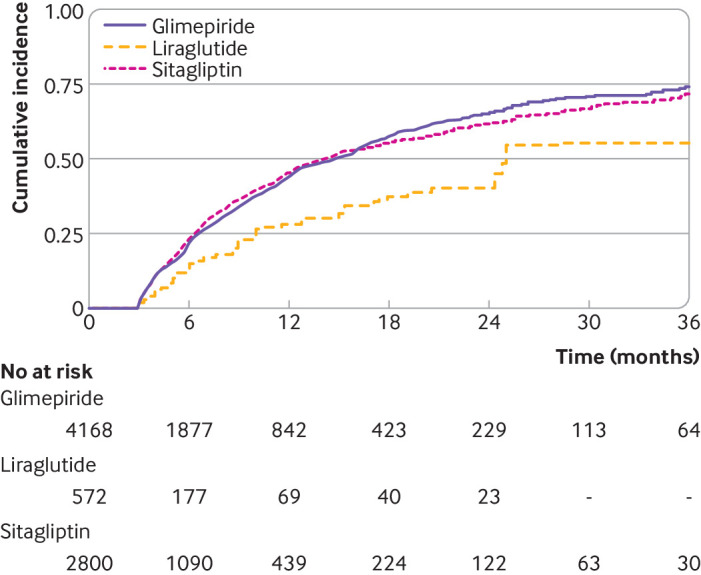
Cumulative incidence rates of primary metabolic failure in propensity score weighted individuals included in the study

Median follow-up until per protocol censoring was 238 days (95% confidence interval 226 to 255 days) in the glimepiride arm, 124 (100 to 150) days in the liraglutide arm, and 186 (179 to 201) days in the sitagliptin arm (see supplemental figure S4). Mean HbA_1c_ decreased most in the liraglutide arm and least in the sitagliptin arm, with differences most pronounced between months 3 and 6 of treatment (fig 1). The median times to primary metabolic failure were 442 days (95% confidence interval 394 to 480 days) in the glimepiride arm, 764 (741 to not calculable) days in the liraglutide arm, and 427 (380 to 483) days in the sitagliptin arm (fig 2). Liraglutide was associated with lower risk of primary metabolic failure compared with glimepiride (hazard ratio 0.57, 95% confidence interval 0.43 to 0.75) and sitagliptin (0.55, 0.41 to 0.73); table 2. No significant difference was observed between sitagliptin and glimepiride (1.03, 0.94 to 1.13). By one year, the estimated cumulative incidence rates of primary metabolic failure were 0.28 (95% confidence interval 0.19 to 0.36) in the liraglutide arm, 0.44 (0.42 to 0.46) in the glimepiride arm, and 0.46 (0.43 to 0.48) in the sitagliptin arm (table 3). These trends in cumulative incidence rates of primary metabolic failure persisted at two years.

**Table 2:** Hazard ratios for primary and secondary metabolic outcomes

Outcomes	Hazard ratio (95% CI)	P value*
**Primary metabolic failure (HbA_1c_ ≥7%)**		
Liraglutide *v* glimepiride	0.57 (0.43 to 0.75)	<0.001
Sitagliptin *v* glimepiride	1.03 (0.94 to 1.13)	0.48
Liraglutide *v* sitagliptin	0.55 (0.41 to 0.73)	<0.001
**Secondary metabolic failure (HbA_1c_ >7.5%)**		
Liraglutide *v* glimepiride	0.61 (0.43 to 0.87)	0.01
Sitagliptin *v* glimepiride	1.04 (0.91 to 1.18)	0.60
Liraglutide *v* sitagliptin	0.59 (0.41 to 0.85)	0.01
**Initiating insulin**		
Liraglutide *v* glimepiride	1.49 (0.76 to 2.93)	0.75
Sitagliptin *v* glimepiride	1.10 (0.77 to 1.58)	0.81
Liraglutide *v* sitagliptin	1.35 (0.67 to 2.72)	0.81

CI=confidence interval; HbA_1c_=hemoglobin A_1c_.

* Adjusted using Holm method.

**Table 3:** Cumulative incidence rates of primary and secondary metabolic failure by treatment arm

	Event rate (95% CI)	
	1 year	2 years
**Primary metabolic failure (HbA_1c_ ≥7%)**		
Glimepiride	0.44 (0.42 to 0.46)	0.65 (0.63 to 0.68)
Liraglutide	0.28 (0.19 to 0.36)	0.40 (0.29 to 0.49)
Sitagliptin	0.46 (0.43 to 0.48)	0.62 (0.58 to 0.65)
**Secondary metabolic failure (HbA_1c_ >7.5%)**		
Glimepiride	0.20 (0.19 to 0.22)	0.37 (0.34 to 0.39)
Liraglutide	0.11 (0.06 to 0.17)	0.17 (0.10 to 0.25)
Sitagliptin	0.22 (0.19 to 0.24)	0.37 (0.33 to 0.41)

CI=confidence interval; HbA_1c_=hemoglobin A_1c_.

### Secondary metabolic failure (HbA_1c_ >7.5%)

Time to secondary metabolic failure was longest in the liraglutide arm (see supplemental figure S5). Liraglutide was associated with lower risk of secondary metabolic failure compared with glimepiride (0.61, 0.43 to 0.87) and sitagliptin (0.59, 0.41 to 0.85); table 2. By one year, the estimated cumulative incidence rates of secondary metabolic failure were 0.11 (95% confidence interval 0.06 to 0.17) in the liraglutide arm, 0.20 (0.19 to 0.22) in the glimepiride arm, and 0.22 (0.19 to 0.24) in the sitagliptin arm (table 3). The difference in event rates persisted at two years.

### Other secondary outcomes

Insulin was started by 84 of 4168 (2.0%) people in the glimepiride arm, 11 of 572 (1.9%) in the liraglutide arm, and 50 of 2800 (1.8%) in the sitagliptin arm, with no significant difference among the three groups (hazard ratios for pairwise comparisons are shown in table 2). Overall, 37 patients experienced emergency department visits or hospital admissions for hypoglycemia during the study period, including <11 in the liraglutide and sitagliptin arms, precluding statistical analyses.

Heart failure, end stage kidney disease, pancreatitis, pancreatic cancer, thyroid cancer, and all cause mortality could not be analyzed owing to too few (<11) events in all treatment groups (supplemental table S11 presents the event rates). No statistically significant differences were observed between groups for major adverse cardiovascular events, retinopathy, neuropathy, other cardiovascular events, cancer, and all cause admissions to hospital (see supplemental table S12).

### Subgroup analyses

Liraglutide was associated with lower risk of primary metabolic failure compared with glimepiride (hazard ratio 0.59, 95% confidence interval 0.44 to 0.78) and sitagliptin (0.58, 0.43 to 0.79) among patients with baseline HbA_1c_ ≥7.0%. No significant differences were observed among the treatment groups in individuals with baseline HbA_1c_ <7.0% (see supplemental table S13). Liraglutide was associated with lower risk of primary metabolic failure compared with glimepiride (0.54, 0.42 to 0.71) and sitagliptin (0.58, 0.44 to 0.77) among those aged <65 years. No significant differences were observed among groups in people aged ≥65 years of age. Liraglutide was also associated with lower risks of primary metabolic failure than glimepiride and sitagliptin in women, but not in men, and in white and Hispanic individuals, but not in black or Asian individuals. Findings were similar for secondary metabolic failure (see supplemental table S14).

### Sensitivity analyses

Another glucose lowering drug was added before discontinuation of the assigned treatment in 423 of 4168 (10%) people in the glimepiride arm, 237 of 572 (41%) in the liraglutide arm, and 419 of 2800 (15%) in the sitagliptin arm. Sensitivity analyses using the as treated censor approach were consistent with the primary analyses (see supplemental figure S6 and table S15). No significant differences were observed among the treatment groups for the pneumonia falsification endpoint (see supplemental table S16).

## Discussion

### Principal findings

In our emulation of the GRADE trial using real world data from an administrative claims database we found that liraglutide was statistically significantly more effective at maintaining glycemic control, defined by time to HbA_1c_ ≥7.0% (primary metabolic failure) and HbA_1c_ >7.5% (secondary metabolic failure) than either glimepiride or sitagliptin. These differences are clinically meaningful, with over 40% more patients in control of their HbA_1c_ when treated with liraglutide than when treated with glimepiride or sitagliptin. We were unable to include insulin glargine in the comparisons because of the small number of individuals treated with this drug who met the GRADE trial eligibility criteria. This was not surprising as treatment with basal insulin in the clinical context examined by the GRADE trial is outside the standard of care and mainstream practice. Additionally, the analytic framework implemented in this work shows that real world data may be an important complement to prospective trials, allowing for efficient and timely examination of pressing clinical questions and inquiries of comparative effectiveness and safety.

Our efforts to emulate all specifications of the GRADE trial were hindered because study conditions are not adequately represented in real world practice as they are not supported by clinical practice guidelines. Although all four study drugs were frequently used by the OLDW population, 80% of adults starting these drugs had to be excluded because they did not meet the prespecified eligibility criteria for the GRADE trial. Nevertheless, this proportion of included participants is still higher than the 9.1% generalizability estimated by the GRADE trial team compared with the overall US population with diabetes.^19^ Most of the people (58.6% overall) were excluded because they did not meet the baseline HbA_1c_ level requirements, including 81.1% of people who started glargine, 71.8% who started liraglutide, 52.8% who started glimepiride, and 51.7% who started sitagliptin. According to current guidelines, the target HbA_1c_ for most non-pregnant adults is 7.0%, such that treatment intensification would not be warranted for some people. Initiation of insulin, in particular, is advised when HbA_1c_ is >9-10%,^14^ ^27^ so starting glargine as a second line drug at HbA_1c_ levels <8.5% would not be consistent with the standard of care^14^ ^27^ or contemporary practice.^28^ ^29^ ^30^ The fact that most people treated with the studied drugs in clinical practice are not represented in the study population raises concerns about the utility and generalizability of the GRADE trial’s findings and its impact on diabetes management, underscoring the important complementary insights that can be gleaned from analyses of real world data (which can be designed to use more pragmatic and generalizable eligibility criteria) as adjuncts to randomized controlled trials.

### Comparison with other studies

We met our objective to conduct all analyses before publication of the GRADE trial findings, and it will be important to ultimately compare our findings with those of the GRADE trial. The greater effectiveness of liraglutide compared with both glimepiride and sitagliptin is consistent with previous studies.^29^ ^31^ ^32^ ^33^ Additionally, subgroup analyses showing greater effectiveness of liraglutide among people with raised baseline HbA_1c_ and in younger patients, generated important hypotheses about the optimal use of liraglutide (and potentially other glucagon-like peptide-1 receptor agonists) in clinical practice to be explored in future research. When the GRADE trial was conceived, drugs’ ability to lower HbA_1c_ was at the forefront of clinical decision making when choosing glucose lowering treatment. Similarly, the sodium-glucose cotransporter-2 inhibitors class of glucose lowering drugs had not yet been incorporated into practice and therefore was excluded as a comparator treatment when the GRADE trial was conceived and designed.

### Strengths and limitations of this study

Our study is strengthened by application of advanced analytic methods that account for measured differences between treatment arms that otherwise confound analyses and preclude causal inference. The generalized boosted based models for the propensity score are more flexible and less sensitive to model misspecification compared with logistic regression. The large and diverse population within OLDW made emulation efforts uniquely possible despite the narrow eligibility criteria specified by the GRADE trial.

Despite rigorous causal inference analytic methods, observational studies are inevitably subject to residual confounding. For the metabolic endpoints, there was evidence of non-proportional hazards, which makes the single summary hazard ratio calculated from the Cox proportional hazards an imperfect estimate for the time varying risk. However, with the goal of emulating the GRADE trial, where the statistical analysis plan was to estimate single summary hazard ratios, we report the same estimate in the emulation. We were also unable to operationalize every component of the GRADE trial’s eligibility criteria and endpoints. For example, we did not require confirmatory HbA_1c_ results to meet the metabolic endpoints and were not able to maintain the same standard timeframe for HbA_1c_ ascertainment as specified in the GRADE trial. Additionally, while the GRADE trial analyses were conducted using the intention-to-treat principle, we a priori chose to use per protocol analysis for the metabolic endpoints because in the absence of randomization, reasons for changing a treatment typically depend on post-initiation factors that could confound the association between the treatment group and the outcome. While advanced statistical methods can account for post-baseline differences between groups in key characteristics, these methods require accurate estimation of the reasons to stop or change treatment, and such estimation is not feasible in this setting using claims data. Duration of follow-up was also different among the treatment arms, which is unavoidable when studying real world practice patterns. In particular, a higher proportion of individuals initiating liraglutide filled only one cycle of treatment before either switching to a different treatment or not refilling their prescription, potentially because of poor tolerability, the need to be administered subcutaneously, or high cost.

Not all people with claims data in OLDW have available laboratory data, as laboratory results are available for a subset of patients based on data sharing agreements between OptumLabs and commercial laboratories. The availability of laboratory results, however, is independent of treatment regimen, and we do not expect it to bias our analyses. The schedule of HbA_1c_ testing in real world practice is contingent on an individual’s current HbA_1c_ level and ability to access care, and on the clinician’s anticipation of changing HbA_1c_ levels. This may have confounded study results by delaying the time to HbA_1c_ reassessment and reaching the study endpoint in people with low baseline HbA_1c_ or with barriers to care. Our evaluation could not account for inclusion and exclusion criteria that could not be operationalized using claims data, including drugs obtained without insurance coverage (eg, obtained through a low cost generic programme,^34^ a patient assistance programme, or a sample), comorbidities that were not coded and billed in a clinical encounter, and information on family history. However, previous studies found the likely number of glucose lowering drugs missing from claims to be low.^35^ Finally, the study cohort comprised people with private and Medicare Advantage health plans, such that results may not fully generalize to people with public health plans or those without insurance coverage.

### Policy implications

Contemporary clinical practice guidelines increasingly focus on the impacts of glucose lowering treatments on hard outcomes that are important to patients beyond HbA_1c_, such as macrovascular and microvascular complications and death,.^36^ Indeed, most recent clinical practice guidelines recommend consideration of glucagon-like peptide-1 receptor agonists and sodium-glucose cotransporter-2 inhibitors even as preferred treatments and independent of the HbA_1c_ level among people at high risk for atherosclerotic cardiovascular disease, kidney disease, and heart failure.^14^ For these outcomes, robust evidence favors liraglutide (of the drug classes examined) in individuals at high risk for atherosclerotic cardiovascular disease,^37^ ^38^ further underscoring the advantage of this drug. It will be important, in future research, to compare the effectiveness of glycemic control achieved by glucagon-like peptide-1 receptor agonists with that of sodium-glucose cotransporter-2 inhibitors, as sodium-glucose cotransporter-2 inhibitors are similarly recommended for people at high risk for cardiovascular disease, kidney disease, and heart failure.^14^

Analytic methods such as those implemented in this study, and in the parallel emulation of PRONOUNCE,^18^ can be leveraged for more timely evaluations of drug effectiveness and safety as long as the treatments being considered are already used in clinical practice. Indeed, work is currently underway to examine the comparative effectiveness of sulfonylurea, glucagon-like peptide-1 receptor agonist, dipeptidyl-peptidase 4 inhibitor, and sodium-glucose cotransporter-2 inhibitor drugs for atherosclerotic cardiovascular disease and other hard outcomes among people at moderate risk for atherosclerotic cardiovascular disease using observational data from real world practice.^39^

### Conclusions

Better understanding of the comparative effectiveness and safety of second line glucose lowering drugs is urgently needed to inform shared decision making in diabetes. Ultimately, the population included in this study and our findings should be compared with those of the GRADE trial, once published in peer reviewed literature, to assess the fidelity and generalizability of results and to improve our understanding of the use of real world data to emulate clinical trials.

**What is already known on this topic**

Real world data are an important source of information about clinical practice, comparative effectiveness and safety, and health outcomes
Such data also have the potential to generate timely, pragmatic evidence on medical treatments as a complement to prospective clinical trials
Multiple classes of second line glucose lowering drugs have been approved for the management of type 2 diabetes, with limited evidence about their comparative effectiveness for glycemic control

**What this study adds**

This study emulated the Glycemia Reduction Approaches in Diabetes: A Comparative Effectiveness Study (GRADE) randomized clinical trial using data from a US administrative claims database to identify the strengths and limitations of using real world data to emulate prospective comparative effectiveness trials, particularly when examining drugs in contexts that may not be the standard of care
Liraglutide was found to be more effective than glimepiride and sitagliptin at lowering glycated hemoglobin (HbA_1c_), supporting its preferential use when substantial glycemic control is needed
Advanced causal inference analytic methods applied to observational data can be used to emulate clinical trials efficiently and effectively

## Data Availability

This study was conducted using deidentified claims data from OptumLabs Data Warehouse. Raw data are not publicly available. The study protocol, code sets, and statistical analysis plan are available online.^40^

## Web extra

Extra material supplied by authors

Supplementary information: Additional methods, tables S1-S16, figures S1-S6, and additional references

## Notes

Contributors: RGM had full access to all the data in the study and takes responsibility for the integrity of the data and the accuracy of the data analysis. RGM is the guarantor. YD analyzed the data and co-drafted the manuscript. ECP co-designed the study, supervised the analyses, and reviewed and edited the manuscript. JDW, SSD, JH, KQ, CG, WC, PN, XY, and TDL provided feedback on study design and reviewed and edited the manuscript. JSR and NDS secured funding, supervised the study, provided feedback on study design, and reviewed and edited the manuscript. RGM co-drafted the manuscript, co-designed the study, and supervised the analyses. The corresponding author attests that all listed authors meet authorship criteria and that no others meeting the criteria have been omitted.

The lead author (RGM) affirms that the manuscript is an honest, accurate, and transparent account of the study being reported; that no important aspects of the study have been omitted; and that any discrepancies from the study as planned (and, if relevant, registered) have been explained.

Dissemination to participants and related patient and public communities: Study results will be disseminated to patient and public communities through peer reviewed publication, reporting of study results to the Food and Drug Administration, and sharing of the results on social media.

Provenance and peer review: Not commissioned; externally peer reviewed.

## References

[1] CDC. National Diabetes Statistics Report Atlanta, Georgia, U.S.A.: U.S. Department of Health and Human Services, Centers for Disease Control and Prevention; 2021 (updated 29 December 2021; cited 26 July 2022]. https://www.cdc.gov/diabetes/data/statistics-report/.

[2] Saeedi P Petersohn I Salpea P IDF Diabetes Atlas Committee . Global and regional diabetes prevalence estimates for 2019 and projections for 2030 and 2045: Results from the International Diabetes Federation Diabetes Atlas, 9^th^ edition. Diabetes Res Clin Pract 2019;157:107843. 10.1016/j.diabres.2019.107843. 31518657

[3] Hemmingsen B Lund SS Gluud C . Intensive glycaemic control for patients with type 2 diabetes: systematic review with meta-analysis and trial sequential analysis of randomised clinical trials. BMJ 2011;343:d6898. 10.1136/bmj.d6898. 22115901 PMC3223424

[4] Rodriguez-Gutierrez R Gonzalez-Gonzalez JG Zuñiga-Hernandez JA McCoy RG . Benefits and harms of intensive glycemic control in patients with type 2 diabetes. BMJ 2019;367:l5887. 10.1136/bmj.l5887. 31690574

[5] American Diabetes Association Standards of Medical Care in Diabetes—2020. Section 6. Glycemic Targets. Diabetes Care 2020;43(Supplement 1):S66-76. 10.2337/dc20-S006. 31862749

[6] Schmittdiel JA Uratsu CS Karter AJ . Why don’t diabetes patients achieve recommended risk factor targets? Poor adherence versus lack of treatment intensification. J Gen Intern Med 2008;23:588-94. 10.1007/s11606-008-0554-8. 18317847 PMC2324158

[7] Shichiri M Kishikawa H Ohkubo Y Wake N . Long-term results of the Kumamoto Study on optimal diabetes control in type 2 diabetic patients. Diabetes Care 2000;23(Suppl 2):B21-9. 10860187

[8] Turner RC Cull CA Frighi V Holman RR UK Prospective Diabetes Study (UKPDS) Group . Glycemic control with diet, sulfonylurea, metformin, or insulin in patients with type 2 diabetes mellitus: progressive requirement for multiple therapies (UKPDS 49). JAMA 1999;281:2005-12. 10.1001/jama.281.21.2005. 10359389

[9] Stratton IM Adler AI Neil HA . Association of glycaemia with macrovascular and microvascular complications of type 2 diabetes (UKPDS 35): prospective observational study. BMJ 2000;321:405-12. 10.1136/bmj.321.7258.405 10938048 PMC27454

[10] Holman RR Paul SK Bethel MA Matthews DR Neil HA . 10-year follow-up of intensive glucose control in type 2 diabetes. N Engl J Med 2008;359:1577-89. 10.1056/NEJMoa0806470. 18784090

[11] NICE. National Institute for Health and Care Excellence Pathways: Managing Blood Glucose in Adults with Type 2 Diabetes: National Institute for Health and Care Excellence; 2019 (updated 26 March 2019; cited 23 April 2019). https://pathways.nice.org.uk/pathways/type-2-diabetes-in-adults.

[12] Garber AJ Abrahamson MJ Barzilay JI . Consensus Statement By The American Association Of Clinical Endocrinologists And American College Of Endocrinology On The Comprehensive Type 2 Diabetes Management Algorithm – 2019 Executive Summary. Endocr Pract 2019;25:69-100. 10.4158/CS-2018-0535. 30742570

[13] Conlin PR Colburn J Aron D Pries RM Tschanz MP Pogach L . Synopsis of the 2017 U.S. Department of Veterans Affairs/U.S. Department of Defense Clinical Practice Guideline: Management of Type 2 Diabetes Mellitus. Ann Intern Med 2017;167:655-63. 10.7326/M17-1362. 29059687

[14] American Diabetes Association Standards of Medical Care in Diabetes—2022. Section 9. Pharmacologic Approaches to Glycemic Treatment. Diabetes Care 2021;45(Supplement_1):S125-43. 10.2337/dc22-S009.

[15] Fang M Wang D Coresh J Selvin E . Trends in Diabetes Treatment and Control in U.S. Adults, 1999-2018. N Engl J Med 2021;384:2219-28. 10.1056/NEJMsa2032271. 34107181 PMC8385648

[16] Nathan DM Buse JB Kahn SE GRADE Study Research Group . Rationale and design of the glycemia reduction approaches in diabetes: a comparative effectiveness study (GRADE). Diabetes Care 2013;36:2254-61. 10.2337/dc13-0356. 23690531 PMC3714493

[17] ClinicalTrials.gov. A Comparative Effectiveness Study of Major Glycemia-lowering Medications for Treatment of Type 2 Diabetes (GRADE). ClinicalTrials.gov Identifier: NCT01794143: U.S. National Library of Medicine. National Institutes of Health. Department of Health and Human Services; 2021 (updated 30 June 2021; cited 7 September 2021). https://clinicaltrials.gov/ct2/show/NCT01794143

[18] Wallach JD Deng Y McCoy RG . Real-world Cardiovascular Outcomes Associated With Degarelix vs Leuprolide for Prostate Cancer Treatment. JAMA Netw Open 2021;4:e2130587. 10.1001/jamanetworkopen.2021.30587. 34677594 PMC8536955

[19] Wexler DJ Krause-Steinrauf H Crandall JP GRADE Research Group . Baseline Characteristics of Randomized Participants in the Glycemia Reduction Approaches in Diabetes: A Comparative Effectiveness Study (GRADE). Diabetes Care 2019;42:2098-107. 10.2337/dc19-0901. 31391203 PMC6804613

[20] Wallace PJ Shah ND Dennen T Bleicher PA Crown WH . Optum Labs: building a novel node in the learning health care system. Health Aff (Millwood) 2014;33:1187-94. 10.1377/hlthaff.2014.0038. 25006145

[21] OptumLabs. OptumLabs and OptumLabs Data Warehouse (OLDW) Descriptions and Citation. Cambridge, MA: May 2019. Reproduced with permission from OptumLabs., 2019.

[22] Benchimol EI Smeeth L Guttmann A RECORD Working Committee . The REporting of studies Conducted using Observational Routinely-collected health Data (RECORD) statement. PLoS Med 2015;12:e1001885. 10.1371/journal.pmed.1001885. 26440803 PMC4595218

[23] McCaffrey DF Griffin BA Almirall D Slaughter ME Ramchand R Burgette LF . A tutorial on propensity score estimation for multiple treatments using generalized boosted models. Stat Med 2013;32:3388-414. 10.1002/sim.5753. 23508673 PMC3710547

[24] Xu S Ross C Raebel MA Shetterly S Blanchette C Smith D . Use of stabilized inverse propensity scores as weights to directly estimate relative risk and its confidence intervals. Value Health 2010;13:273-7. 10.1111/j.1524-4733.2009.00671.x. 19912596 PMC4351790

[25] Austin PC . Balance diagnostics for comparing the distribution of baseline covariates between treatment groups in propensity-score matched samples. Stat Med 2009;28:3083-107. 10.1002/sim.3697. 19757444 PMC3472075

[26] Korn EL . Censoring distributions as a measure of follow-up in survival analysis. Stat Med 1986;5:255-60. 10.1002/sim.4780050306. 3738291

[27] American Diabetes Association Standards of Medical Care in Diabetes—2022. Section 6. Glycemic Targets. Diabetes Care 2021;45(Supplement_1):S83-96. 10.2337/dc22-S006.

[28] Liss DT Kang RH Lancki N . Costs for commercially insured adults prescribed second-line diabetes medications. Am J Manag Care 2021;27:e72-9. 10.37765/ajmc.2021.88601. 33720672

[29] Khunti K Charbonnel B Cooper A DISCOVER investigators . Associations between second-line glucose-lowering combination therapies with metformin and HbA1c, body weight, quality of life, hypoglycaemic events and glucose-lowering treatment intensification: The DISCOVER study. Diabetes Obes Metab 2021;23:1823-33. 10.1111/dom.14400. 33852202

[30] Nicolucci A Charbonnel B Gomes MB . Treatment patterns and associated factors in 14 668 people with type 2 diabetes initiating a second-line therapy: Results from the global DISCOVER study programme. Diabetes Obes Metab 2019;21:2474-85. 10.1111/dom.13830. 31297947 PMC6852520

[31] Tsapas A Avgerinos I Karagiannis T . Comparative Effectiveness of Glucose-Lowering Drugs for Type 2 Diabetes: A Systematic Review and Network Meta-analysis. Ann Intern Med 2020;173:278-86. 10.7326/M20-0864. 32598218

[32] Tran S Retnakaran R Zinman B Kramer CK . Efficacy of glucagon-like peptide-1 receptor agonists compared to dipeptidyl peptidase-4 inhibitors for the management of type 2 diabetes: A meta-analysis of randomized clinical trials. Diabetes Obes Metab 2018;20(Suppl 1):68-76. 10.1111/dom.13137. 29364587

[33] Palmer SC Mavridis D Nicolucci A . Comparison of Clinical Outcomes and Adverse Events Associated With Glucose-Lowering Drugs in Patients With Type 2 Diabetes: A Meta-analysis. JAMA 2016;316:313-24. 10.1001/jama.2016.9400. 27434443

[34] Pauly NJ Brown JD . Prevalence of Low-Cost Generic Program Use in a Nationally Representative Cohort of Privately Insured Adults. J Manag Care Spec Pharm 2015;21:1162-70. 10.18553/jmcp.2015.21.12.1162. 26679965 PMC10398242

[35] Rowan CG Flory J Gerhard T . Agreement and validity of electronic health record prescribing data relative to pharmacy claims data: A validation study from a US electronic health record database. Pharmacoepidemiol Drug Saf 2017;26:963-72. 10.1002/pds.4234. 28608510

[36] Rodriguez-Gutierrez R McCoy RG . Measuring What Matters in Diabetes. JAMA 2019;321:1865-6. 10.1001/jama.2019.4310. 30985865 PMC7332201

[37] Mann JFE Ørsted DD Brown-Frandsen K LEADER Steering Committee and Investigators . Liraglutide and Renal Outcomes in Type 2 Diabetes. N Engl J Med 2017;377:839-48. 10.1056/NEJMoa1616011. 28854085

[38] Marso SP Daniels GH Brown-Frandsen K LEADER Steering Committee LEADER Trial Investigators . Liraglutide and Cardiovascular Outcomes in Type 2 Diabetes. N Engl J Med 2016;375:311-22. 10.1056/NEJMoa1603827. 27295427 PMC4985288

[39] PCORI. Observational Analyses of Second-Line Pharmacological Agents in Type 2 Diabetes Washington, DC: Patient-Centered Outcomes Research Institute; 2021 (cited 7 September 2021). https://www.pcori.org/funding-opportunities/announcement/observational-analyses-second-line-pharmacological-agents-type-2-diabetes-cycle-2-2020.

[40] Yale University-Mayo Clinic CERSI. 2021 (cited 29 September 2021]. https://medicine.yale.edu/core/current_projects/cersi/research/.

