# Supplementary material for: Emulating the GRADE trial using real world data: retrospective comparative effectiveness study
Source: BMJ. 2022 Oct 3;379:e070717. doi: 10.1136/bmj-2022-070717 (PMC9527635; doi:10.1136/bmj-2022-070717)
Supplement: Supplementary file 1 — Supplementary information: Additional methods, tables S1-S16, figures S1-S6, and additional references [file deny070717.ww.pdf]

Online Only Supplemental Material

Contents

Supplemental Methods 2
Source data for individual-level socioeconomic information 2
Propensity score estimation with multiple treatments using generalized boosted models 2
Figure S1. Cohort creation prior to application of GRADE Trial eligibility criteria 3
Table S1. Eligibility criteria for the GRADE trial and its OLDW emulation. 4
Table S2. Code sets used to ascertain comorbidities included in eligibility criteria, baseline covariates, or outcomes 6
Table S3. Medications included in eligibility criteria or baseline covariates. 10
Table S4. Frequency and timing of HbA_{1c} tests in each study arm. 11
Table S5. HbA_{1c} test intervals in each study arm, stratified by baseline HbA_{1c} level. 12
Table S6. Secondary outcomes included in the analyses. 13
Figure S2. Distribution of stabilized propensity score weights, stratified by drug 15
Figure S3. Censoring approaches 16
Table S7. Patients excluded from analyses due to not meeting GRADE Trial eligibility criteria. 17
Table S8. Complete list of medications included in the final cohort. 18
Table S9. Baseline characteristics of patients in the four treatment arms prior to weighting. 19
Table S10. Pairwise standardized mean difference for weighted cohort 22
Figure S4. Inverse probability of treatment weighted Kaplan Meier curve for the time to right censoring by treatment groups. 23
Figure S5. Cumulative risks of secondary metabolic failure in propensity score weighted patients. 24
Table S11. Rates of secondary microvascular, macrovascular, and safety endpoints (weighted) 25
Table S12. Hazard ratios of secondary microvascular, macrovascular, and safety endpoints 26
Table S13. Subgroup Analyses for Primary Metabolic Failure 27
Table S14. Subgroup Analyses for Secondary Metabolic Failure 29
Figure S6. Cumulative risks of primary and secondary metabolic failure (sensitivity analysis).. 31
Primary metabolic failure 31
Secondary metabolic failure 32
Table S15. Hazard ratios for primary and secondary metabolic failure (sensitivity analysis) 34
Table S16. Falsification end point analysis 34
REFERENCES 35

Supplemental Methods.

Source data for individual-level socioeconomic information
Socioeconomic status data available in OptumLabs Data Warehouse (OLDW) is sourced from a national supplier of consumer marketing data, which includes consumer-specific demographic, behavioral, and lifestyle information. Much like administrative claims, the data have been collected for purposes other than research and variable imputation is conducted by the Data Supplier as appropriate. Socioeconomic variables included in our analyses are race, ethnicity, and annual household income. Race and ethnicity are imputed based on a model run by the Data Supplier using an individual's name (first, last, middle) and geographic location, then categorized into five race values in OLDW: W (White), B (Black), H (Hispanic), A (Asian), and U (Unknown). The Data Supplier imputes estimated household income based on a model using both public and private consumer data. This variable is assigned at the household level where a "household" is defined as individuals with the same surname living at the same street address.

Propensity score estimation with multiple treatments using generalized boosted models
Let $T_i \in \{1, 2, \ldots, M\}$ indicate the treatment group for patient i out of the M treatment groups, and X_i be the collection of baseline covariates. Then the propensity score can be defined as $\mathrm{ps_t} = \Pr(T = t \mid X)$. For each treatment group, a logistic regression model was estimated for the given treatment versus all others. Regular logistic regression could be used to estimate the individual propensity scores, but requires the functional form of the baseline covariates in the regression model to be close to the truth. An alternative is to estimate the functional form of the baseline covariates using a boosting model as described in McCaffrey et al (2013).[1] The collection of generalized boosted models was estimated using the *gbm* package in R.[2] The base model in the boosted ensemble was a regression tree with interaction depth 3. The optimal number of trees in the boosted ensemble was selected based on the number of trees that minimized the maximum standardized effect size between treatment groups across the baseline covariates.

Figure S1. Cohort creation prior to application of GRADE Trial eligibility criteria.

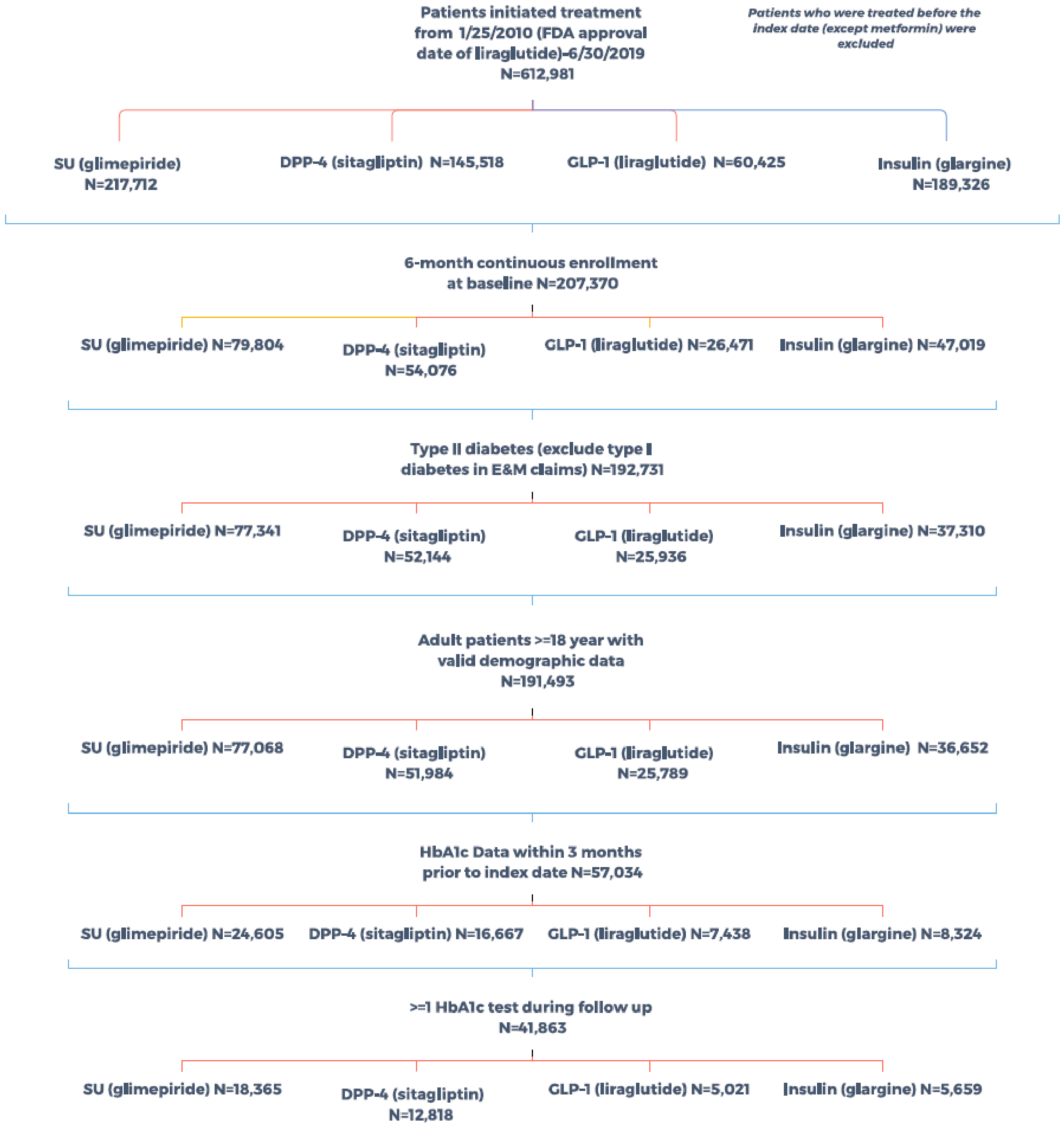

Table S1. Eligibility criteria for the GRADE trial and its OLDW emulation.

GRADE Eligibility Criteria	Operational Definition in OLDW
**Inclusion criteria**	
Men or women ≥30 years of age at time of diabetes diagnosis.<br>For American Indians: ≥20 years of age at time of diagnosis	Age ≥30 years at index date (*American Indians are classified as "other" race/ethnicity in OLDW*)
Duration of diagnosed diabetes: <10 years determined as accurately as possible on the basis of available records at screening	N/A (*We are not able to determine the duration of diagnosed diabetes*)
HbA_{1c} criteria (at final run-in visit, ~2 weeks before randomization): 6.8–8.5% (51–69 mmol/ mol)	HbA_{1c} result in the range (6.8–8.5%) within 3 months (inclusive) of the index date
Taking a daily dose of ≥1,000 mg metformin for a minimum of 8 weeks at final run-in	Adherent to metformin (no dose requirement) for ≥ 8 weeks before the index date
Willingness to administer daily subcutaneous injections, take a second diabetes drug after randomization, potentially initiate insulin, intensify insulin therapy if study metabolic goals are not met, and perform self-monitoring of blood glucose	N/A
A negative pregnancy test for all women of childbearing potential (i.e., premenopausal, not surgically sterile)	No diagnosis or procedure codes at any position for pregnancy within 6 months before and inclusive of the index date
Provision of signed and dated informed consent before any study procedures	N/A
**Exclusion criteria**	
Suspected type 1 diabetes (lean with polyuria, polydipsia, and weight loss with little response to metformin) or secondary diabetes resulting from specific causes (e.g., previously diagnosed monogenic syndromes, pancreatic surgery, pancreatitis)	Diagnosis codes for type 1 diabetes at any position of any E&M encounter at any time during enrollment period prior index date<br>*(Excluded as part of inclusion criteria)*
Current or previous (within past 6 months) treatment with any diabetes drug or glucose lowering medication other than metformin, including short-term insulin use during hospitalization	Fill of any diabetes medications (except metformin) within 6 months before index date<br>*(Excluded as part of inclusion criteria)*
More than 10 years of treatment with metformin at time of randomization	N/A
History of intolerance, allergy, or other contraindications to any of the proposed study medications	N/A
A life-threatening event within 30 days before screening or currently planned major surgery	N/A
Any major cardiovascular event in previous year, including history of myocardial infarction, stroke, or vascular procedure, such as coronary artery or peripheral bypass grafting, stent placement (peripheral or coronary), or angioplasty	Diagnosis and procedure codes at any position for major cardiovascular events within one year before and including the index date
Plans for pregnancy during the course of the study for women of childbearing potential	Diagnosis and procedure codes at any position for pregnancy within 6 months before and inclusive of the index date<br>*(Excluded as part of inclusion criteria)*

GRADE Eligibility Criteria	Operational Definition in OLDW
History of or planning for bariatric surgery, including banding procedures or surgical gastric and/or intestinal bypass	Diagnosis and procedure codes at any position for bariatric surgery within 6 months before and including the index date
History of congestive heart failure (New York Heart Association class III or IV)	Diagnosis codes for congestive heart failure at any position within 6 months before and inclusive of the index date
History of conditions that are specific contraindications to any of the study medications	N/A
Serum creatinine level ≥1.4 mg/dL in women and ≥1.5 mg/dL in men or end-stage renal disease requiring renal replacement therapy	Serum creatinine level ≥1.4 mg/dL in women and ≥1.5 mg/dL in men; or kidney transplant codes at baseline; or ≥3 dialysis codes at baseline; or Diagnosis code for ESRD within 6 months before and inclusive of the index date, all codes were pulled at any position
History of cancer, other than nonmelanoma skin cancer, that required therapy in the 5 years before randomization	Diagnosis codes at any position for cancer (except nonmelanoma skin cancer) within 6 months before and inclusive of the index date
Treatment with oral or systemic glucocorticoids (other than short-term treatment, e.g., for poison ivy) or disease likely to require periodic or regular glucocorticoid therapy (inhaled steroids allowed)	Adherent to oral glucocorticoids for ≥3 months at baseline
Treatment with atypical antipsychotics	A prescription of atypical antipsychotics within 3 months before or on the index date
Clinically or medically unstable with expected survival <1 year	N/A

Table S2. Code sets used to ascertain comorbidities included in eligibility criteria, baseline covariates, or outcomes. All baseline covariates were assessed at all positions in diagnosis and procedure codes.

Comorbidity	ICD-9 Codes	ICD-10 codes	CPT codes	Revenue codes
**Bariatric surgery**	V45.86, 43.82, 43.89, 44.38, 44.39, 44.68, 44.95, 44.69	Z98.84, 0DQ64ZZ, 0DQ60ZZ, 0DB80ZZ, 0D16078, 0D16479, 0DV64CZ, 0D190Z9, 0DB60ZZ	43644, 43645, 43770, 43775, 43842, 43843, 43845, 43846, 43847, S2082, S2085	
**Cancer (except non-melanoma skin cancer)**	14x.xx, 15x.xx, 160.x-165.x, 170.x-172.x, 174.x-176.x, 179-189.x, 190.x-195.x, 199.xx-208.xx (except 203.x1, 204.x1, 205.x1, 206.x1, 207.x1, 208.x1)	C00.x-C14.x, C15.x-C26.x, C3x.xx, C40.xx-C41.x, C43.x, C4A.xx, C45.x-C49.xx, C50.xxx, C51.x-C58, C60.x-C63.x, C64.x-C68.x, C69.xx-C72.x, C73-C76.x, C7A.xx, C80.xx-C96.x (except C90.x1, C91.x1, C92.x1, C93.x1, C94.x1, C95.x1)		
**Coronary artery disease**	429.2, 410.x, 411.x, 412.x, 413.x, 414.x	I20.x, I21.x, I22.x, I23.x, I24x, I25.x		
**Coronary artery bypass grafting surgery, percutaneous coronary intervention, lower extremity revascularization**	36.1x, V45.81, 414.02, 414.03, 414.04, 414.05<br><br>V45.82, 36.0x, 00.66<br><br>39.25, 39.29, 38.08, 38.16, 38.18, 38.38, 38.48, 38.68, 38.88, 3950, 39.90, 00.55, 84.3, 84.1x	02100x, 02110x, 02120x, 02130x, Z95.1, T82.21x, I25.7x, I25.810, I25.812<br><br>Z98.61, Z95.5, 0270x, 0271x, 0272x, 0273x, 02C0x, 02C1x, 02C2x, 02C3x<br><br>041x, 047x, 04Bx, 04Cx, 04Lx, 04Px, 04Rx (4th letter C-Y, except G, I, O, X)	33510, 33511, 33512, 33513, 33514, 33516, 33517, 33518, 33519, 33521, 33522, 33523, 33533, 33534, 33535, 33536, 4110F<br><br>92920, 92921, 92924, 92925, 92928, 92929, 92933, 92934, 92937, 92938, 92941, 92943, 92944, 92980, 92981, 92982, 92984, 92995, 92996, 92975, 92977	
**Cerebrovascular disease**	430, 431, 432.x, 433.xx, 434.xx 435.x, 436, 437.x, 438.xx, V12.54	G45.0, G45.1, G45.2, G45.8, G45.9, G46.x, I60.xx, I61.x (except I61.0), I62.xx, I63.xxx, I65.xx, I66.xx, I67.8x (except I67.83, I67.84), I67.9, I69.xxx, Z86.73		

Comorbidity	ICD-9 Codes	ICD-10 codes	CPT codes	Revenue codes
**Chronic kidney disease**	403.01, 403.11, 403.91, 404.02, 404.03, 404.12, 404.13, 404.92, 404.93, 585.3, 585.4, 585.5, 585.6, 792.5, 996.81, V42.0, V45.1, V45.11, V45.12, V56, V56.0, V56.1, V56.2, V56.3, V56.31, V56.32, V56.8	I12.0, I13.11, I13.2, I95.3, N18.3, N18.4, N18.5, N18.6, R88.0, T86.1, T86.10, T86.11, T86.12, T86.13, T86.19, Y84.1, Z48.22, Z49, Z49.0, Z49.01, Z49.02, Z49.3, Z49.31, Z49.32, Z91.15, Z94.0, Z99.2, T81502x, T81512x, T81522x, T81532x, T81592x, T85611x, T85621x, T85631x, T85651x, T8571x		
**End-stage kidney disease, dialysis, transplantation**	403.01, 403.11, 403.91, 404.02, 404.03, 404.12, 404.13, 404.92, 404.93, 585.5, 585.6, 792.5, 996.81, V42.0, V45.1, V45.11, V45.12, V56.x, V56.0, V56.1, V56.2, V56.3, V56.31, V56.32, V56.8 39.95, 54.98, 55.53, 55.6, 55.69	I12.0, I13.11, I13.2, I95.3, N185, N186, R88.0, T81.502x, T81.512x, T81.522x, T81.532x, T81.592x, T85.611x, T85.621x, T85.631x, T85.651x, T85.71x, T86.1, T86.10, T86.11, T86.12, T86.13, T86.19, Y84.1, Z48.22, Z49, Z49.0, Z49.01, Z49.02, Z49.3, Z49.31, Z49.32, Z91.15, Z94.0, Z99.2 0TT00ZZ, 0TT04ZZ, 0TT10ZZ, 0TT14ZZ, 0TT30ZZ, 0TT34ZZ, 0TT37ZZ, 0TT38ZZ, 0TT40ZZ, 0TT44ZZ, 0TY00Z0, 0TY00Z1, 0TY00Z2, 0TY10Z0, 0TY10Z1, 0TY10Z2, 3E1M39Z, 5A1D00Z, 5A1D60Z	50340, 50360, 50365, 50370, 90935, 90937, 90940, 90945, 90947, 90957, 90958, 90959, 90960, 90961, 90962, 90965, 90966, 90969, 90970, 90999, G0257, S9335, 99512	0800, 0801, 0802, 0803, 0804, 0805, 0806, 0807, 0808, 0809, 0820, 0821, 0822, 0823, 0824, 0825, 0826, 0827, 0828, 0829, 0830, 0831, 0832, 0833, 0834, 0835, 0836, 0837, 0838, 0839, 0840, 0841, 0842, 0843, 0844, 0845, 0846, 0847, 0848, 0849, 0850, 0851, 0852, 0853, 0854, 0855, 0856, 0857, 0858, 0859, 0880, 0881, 0882, 0883, 0884, 0885, 0886, 0887, 0888, 0889
**Hyperglycemia**	250.10, 250.11, 250.12, 250.13, 250.20, 250.21, 250.22, 250.23	E10.10, E10.11, E11.10, E11.11, E13.10, E13.11, E11.00, E11.01, E13.00, E13.01		
**Heart failure**	398.91, 402.01, 402.11, 402.91, 404.01, 404.03, 404.11, 404.13, 404.91, 404.93, 428.xx	I09.81, I11.0, I13.0, I13.2, I50.xx		

Comorbidity	ICD-9 Codes	ICD-10 codes	CPT codes	Revenue codes
**Hypoglycemia**	251.0, 251.1, 251.2, 962.3, 250.8x (for 250.8x: if no concurrent 259.8, 272.7, 681.xx, 682.xx, 686.9x, 707.1x-707.2x, 707.8, 707.9, 709.3, 730.0x-730.2x, 731.8)	E10.641, E10.649, E11.641, E11.649, E13.641, E13.649, E16.0, E16.1, E16.2, T38.3X1A, T38.3X1D, T38.3X1S, T38.3X2A, T38.3X2D, T38.3X2S, T38.3X3A, T38.3X3D, T38.3X3S, T38.3X4A, T383X4D, T38.3X4S, T38.3X5A, T383X5D, T38.3X5S		
**Myocardial infarction**	410.x, 412.x	I21.x, I22.x, I23.x, I252.x		
**Nephropathy**	593.9, 586, 250.4x, 249.4x, 580.x, 581.x, 582.x, 583.x, 585.x	E08.21, E08.22, E08.29, E09.21, E09.22, E09.29, E10.21, E10.22, E10.29, E11.21, E11.22, E11.29, E13.21, E13.22, E13.29, N19, N00.x, N03.x, N04.x, N05.x, N18.x		
**Neuropathy**	357.2, 337.1, 356.9, 358.1, 458.0, 536.3, 564.5, 596.54, 713.5, 951.0, 951.1, 951.3, 250.6x, 249.6x, 337.0x, 354.x, 355.x	G90.09, G90.8, G90.9, G99.0, G60.9, G73.3, G90.01, I95.1, K31.84, K59.1, N31.9, E08.4x, E09.4x, E10.4x, E11.4x, E13.4x, G56.x, G57.x, H49.x, M14.6x, S04.x		
**Peripheral vascular disease**	442.3, 440.21, 443.81, 443.9, 892.1, 040.0, 444.22, 785.4, 250.7x, 249.7, 707.1x	E08.51, E09.51, E10.51, E11.51, E13.51, E08.59, E09.59, E10.59, E11.59, E13.59, E08.621, E09.621, E10.621, E11.621, E13.621, I72.4, I73.89, I73.9, A48.0, I74.3, I96, E08.52, E09.52, E10.52, E11.52, E13.52, I70.21x, S91.3x, L97.x		
**Pneumonia**	487.0, 480.x, 481.x 482.x, 483.x, 485.x, 486.x	A01.03, A02.22, A37.01, A37.11, A37.81, A37.91, A50.04, A54.84, B01.2, B05.2, B06.81, B77.81, J09.X1, J13, J14, J17, J84.2, J85.1, J85.2, J95.851, J10.0x, J11.0x, J12.x, J15.x, J16.x, J18.x, J84.11x		

Comorbidity	ICD-9 Codes	ICD-10 codes	CPT codes	Revenue codes
**Retinopathy**	362.01, 362.03, 362.04, 362.05, 362.06, 362.07, 362.53, 362.81, 362.82, 362.83, 362.02, 379.23, 250.5x, 249.5x, 362.1x, 361.x, 369.x	H35.9, E08.3x, E09.3x, E10.3x, E11.3x, E13.3x, H35.0x, H35.35x, H35.6x, H35.8x, H33.x, H54.x, H43.1x		
**Stroke**	433.01, 433.11, 433.21, 433.31, 433.81, 433.91, 434.01, 434.11, 434.91, 436, 430, 431	I63.x, I60.x, I61.x, I69.0x, I69.1x, I69.3x		
**Type 1 diabetes**	250.x1, 250.x3	E10.x		

Table S3. Medications included in eligibility criteria or baseline covariates.

Medication Class	Included Medications
**Anti-psychotics**	Aripiprazole, Asenapine, Asenapine Maleate, Brexpiprazole, Cariprazine HCL, Clozapine, Lurasidone HCL, Olanzapine, Olanzapine/Fluoxetine HCL, Paliperidone, Quetiapine Fumarate, Risperidone, Ziprasidone HCL
**ACE inhibitors**	Benazepril HCL, Captopril, Enalapril Maleate, Fosinopril Sodium, Lisinopril, Perindopril Erbumine, Quinapril HCL, Ramipril, Trandolapril, Moexipril HCL, Benazepril/Hydrochlorothiazide, Captopril/Hydrochlorothiazide, Enalapril/Hydrochlorothiazide, Fosinopril/Hydrochlorothiazide, Lisinopril/Hydrochlorothiazide, Moexipril/Hydrochlorothiazide, Quinapril/Hydrochlorothiazide, Amlodipine Besylate/Benazepril, Enalapril Maleate/Diltiazem, Enalapril Maleate/Felodipine, Perindopril Arginine/Amlodipine Bes, Trandolapril/Verapamil HCL
**Angiotensin receptor blockers**	Candesartan Cilexetil, Irbesartan, Losartan Potassium, Olmesartan Medoxomil, Telmisartan, Valsartan, Azilsartan Medoxomil, Eprosartan Mesylate, Azilsartan Med/Chlorthalidone, Olmesartan/Hydrochlorothiazide, Candesartan Cilexetil/HCTZ, Candesartan/Hydrochlorothiazide, Eprosartan Mesylate/HCTZ, Eprosartan/Hydrochlorothiazide, Irbesartan/Hydrochlorothiazide, Losartan Potassium/HCTZ, Losartan/Hydrochlorothiazide, Olmesartan Medoxomil/HCTZ, Olmesartan/Hydrochlorothiazide, Telmisartan/HCTZ, Telmisartan/Hydrochlorothiazide, Valsartan/Hydrochlorothiazide
**Warfarin and direct oral anti-coagulants (DOAC)**	Warfarin, Dabigatran, Rivaroxaban, Apixaban, Edoxaban
**Glucocorticoids**	Hydrocortisone, Cortisone Acetate, Prednisone, Prednisolone, Methylprednisolone, Triamcinolone, Dexamethasone, Betamethasone
**Lipid-lowering medications** (not statins)	Alirocumab, Evolocumab, Ezetimibe, Mipomersen, Lomitapide, Bezafibrate, Clofibrate, Fenofibrate, Fenofibric Acid, Gemfibrozil, Cholestyramine, Colesevelam, Colestipol
**Peripheral neuropathy medications**	Gabapentin, Pregabalin, Duloxetine
**Statins**	Atorvastatin, Rosuvastatin, Simvastatin, Pravastatin, Lovastatin, Fluvastatin, Pitavastatin
**Biguanide**	Metformin
**Sulfonylurea**	Glimepiride, Glipizide, Glyburide
**DPP-4 inhibitor**	Alogliptin, linagliptin, sitagliptin, saxagliptin
**GLP-1 receptor agonist**	Exenatide, liraglutide, albiglutide, dulaglutide, semaglutide, lixisenatide
**SGLT2 inhibitor**	Canagliflozin, empagliflozin, dapagliflozin, ertugliflozin
**Thiazolidinedione**	Pioglitazone, Rosiglitazone
**Basal insulin**	NPH, Isophane, Zinc, Glargine, Detemir, Degludec
**Bolus insulin**	Regular insulin, Beef insulin, Pork insulin, Aspart, Lispro, Glulisine
**Other glucose-lowering medications**	Nateglinide, Repaglinide, Pramlintide, Acarbose, Miglitol

Table S4. Frequency and timing of HbA_{1c} tests in each study arm. Because hemoglobin A_{1c} (HbA_{1c}) testing frequency and intervals vary in real-world practice, and differential availability of test results between study treatment arms may bias the results, we examined the frequency, intervals, and total number of available HbA_{1c} tests within each treatment arm. Importantly, clinical practice guidelines advise that testing frequency is determined by the patient's glycemic control (i.e., HbA_{1c} level and glycemic variability) rather than their treatment regimen.[3]

Medication	N	Mean	Median	Lower Quartile	Upper Quartile	Min	Max
**Days between HbA_{1c} testing**							
Glimepiride	4318	156	123	92	186	1	2196
Liraglutide	690	155	119	90	184	1	1619
Sitagliptin	2993	158	121	92	188	1	2098
**Days between index date and first HbA_{1c} after the index date**							
Glimepiride	4318	161	116	86	184	1	2196
Liraglutide	690	149	96	72	174	1	1497
Sitagliptin	2993	157	106	81	182	1	2098
**Number of HbA_{1c} tests after the index date, before censoring per protocol**							
Glimepiride	3017	2	2	1	4	1	27
Liraglutide	387	2	1	1	3	1	23
Sitagliptin	1898	2	2	1	3	1	20
**Number of HbA_{1c} tests after the index date, before censoring per intention to treat**							
Glimepiride	4318	4	3	2	6	1	33
Liraglutide	690	3	3	2	6	1	29
Sitagliptin	2993	4	3	2	6	1	33

Table S5. HbA_{1c} test intervals in each study arm, stratified by baseline HbA_{1c} level. Because hemoglobin A_{1c} (HbA_{1c}) testing frequency is informed by the patient's baseline HbA_{1c} level,[3] we examined the number of days in between sequential HbA_{1c} results in each treatment arm stratified by their baseline HbA_{1c}.

Medication	Baseline HbA_{1c}	N	Mean	Median	Lower Quartile	Upper Quartile
Glimepiride	<7.0%	295	150	121	91	185
	≥7.0%	4023	156	123	92	187
Liraglutide	<7.0%	100	174	135	91	217
	≥7.0%	590	152	118	90	182
Sitagliptin	<7.0%	289	163	120	91	190
	≥7.0%	2704	158	121	92	188

Table S6. Secondary outcomes included in the analyses.

1) ***Metabolic endpoints***
   (1) Proportion of subjects among those randomized to each treatment that has reached the primary metabolic outcome over time
   (2) Time to secondary metabolic failure (HbA_{1c} >7.5%) after having reached the primary outcome, while receiving the assigned regimen.
   (3) Proportion of subjects among those randomized to each treatment that has reached secondary metabolic failure over time.
   (4) Time to the need for insulin therapy while being treated with the assigned regimen.
   (5) Proportion of subjects among those randomized to each treatment group that has initiated insulin therapy over time.
   (6) Number of emergency department visits and/or hospitalizations for hypoglycemia per 1000 patients per year, ascertained using primary diagnosis codes in ED/hospitalization claims

2) ***Microvascular outcomes***
   (1) Incidence* of end-stage kidney disease, ascertained using diagnosis and procedure codes at any position in E&M claims (note: this outcome was not present in GRADE, which examined progression of microalbuminuria or worsening of eGFR)
   (2) Incidence of diabetic retinopathy or documentation of retinal photocoagulation for diabetic retinopathy, ascertained using diagnosis and procedure codes at any position in E&M claims.
   (3) Incidence of peripheral neuropathy, ascertained using diagnosis codes at any position in E&M claims.

3) ***Cardiovascular outcomes***
   (1) Incidence of major adverse cardiovascular events (MACE: death, nonfatal myocardial infarction, nonfatal stroke), MI and stroke were ascertained using primary and first secondary diagnosis codes in hospitalization claims. Death was ascertained from OLDW, where it is derived from the Social Security Administration Death Master File, deceased status from electronic medical records, death as a reason for disenrollment, death indicated by inpatient discharge status, obituary information, and Medicare Advantage beneficiary report information.
   (2) Incidence of other cardiovascular events including unstable angina requiring hospitalization or revascularization, ascertained using primary and first secondary diagnosis and procedure codes in hospitalization claims
   (3) Incidence of congestive heart failure requiring hospitalization, ascertained using primary diagnosis codes in hospitalization claims.

5) ***Safety outcomes (adverse events)***
   (1) Incidence of pancreatitis, ascertained using diagnosis codes at any position in ED/hospitalization claims
   (2) Incidence of pancreatic and medullary thyroid cancer, ascertained using diagnosis codes at any position in E&M claims

(3) Incidence of cancer (except non-melanoma skin cancer) , ascertained using diagnosis codes at any position in E&M claims

6) ***Other outcomes***

(1) All-cause mortality
(2) Any hospital admission, ascertained using revenue codes

*Incidence: no codes at any position for this category in the past 12 months

Figure S2. Distribution of stabilized propensity score weights, stratified by drug.

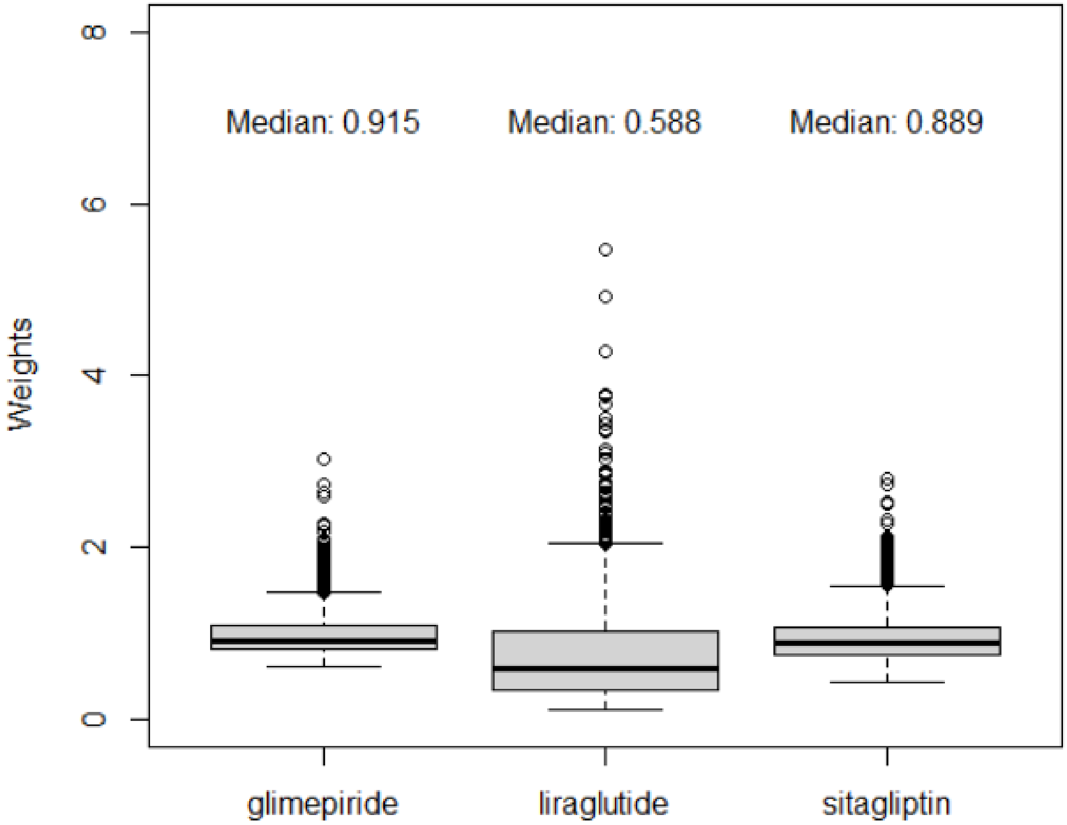

Figure S3. Censoring approaches. For the primary outcome (primary metabolic failure) and the secondary outcomes of secondary metabolic failure and initiation of insulin, patients were censored when the assigned treatment drug was discontinued (point 3) or at death, disenrollment, or end of the study period (point 4), whichever came first. For all other secondary outcomes, we censored at the time of death, disenrollment, or end of the study period (point 4). As a sensitivity analysis ("as treated approach"), patients were censored when they added a different medication class (point 2), discontinued the assigned drug (point 3), or at death, disenrollment, or end of the study period (point 4), whichever came first.

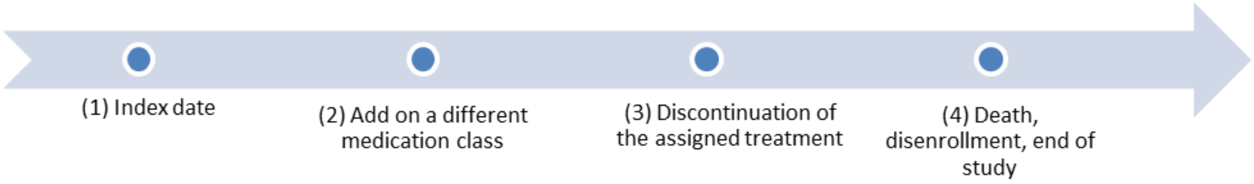

Rationale: For the primary analysis, a patient was right censored if they discontinued their initial study treatment ("per protocol" approach), but we did not consider adding an additional antidiabetic treatment to the current initiated treatment as sufficient to censor the individual from follow-up. Since the primary outcome defined in the GRADE protocol was "time to an HbA_{1c} value ≥7% while subjects are treated with the maximally tolerated doses of both metformin and the randomly assigned medication," we similarly continued follow patients as long as they were still taking their initiated study treatment. However, because adding an additional treatment may indicate a different clinical course, a sensitivity analysis was performed to censor at these time points to evaluate sensitivity of the treatment group comparisons ("as treated" approach).

Table S7. Patients excluded from analyses due to not meeting GRADE Trial eligibility criteria. GRADE eligibility criteria were applied to the base population of adults (age ≥18 years) with type 2 diabetes included in OptumLabs Data Warehouse (OLDW) who had available hemoglobin A_{1c} data and the requisite baseline enrollment period.

GRADE Eligibility Criteria using the Operational Definition in OLDW	Overall (N =41,863)	Glargine (N =5659)	Glimepiride (N =18365)	Liraglutide (N =5021)	Sitagliptin (N =12,818)
Total % of patients eligible for GRADE	8252 (19.7%)	251 (4.4%)	4318 (23.5%)	690 (13.7%)	2993 (23.3%)
Total % of patients ineligible for GRADE	33611 (80.3%)	5408 (95.6%)	14047 (76.5%)	4331 (86.3%)	9825 (76.7%)
**Patients who did NOT meet the inclusion criterion below (%)**					
Age ≥30 years at index date	473 (1.1%)	143 (2.5%)	124 (0.7%)	135 (2.7%)	71 (0.6%)
Have HbA_{1c} between 6.8–8.5% within 3 months of index date	24529 (58.6%)	4591 (81.1%)	9701 (52.8%)	3606 (71.8%)	6631 (51.7%)
Adherent to metformin for ≥8 weeks before the index date	20376 (48.7%)	3874 (68.5%)	7997 (43.5%)	2749 (54.8%)	5756 (44.9%)
No diagnosis or procedure codes for pregnancy at baseline	98 (0.2%)	56 (1.0%)	12 (0.1%)	19 (0.4%)	11 (0.1%)
**Patients who met one of the following exclusion criteria (%)**					
Diagnosis and procedure codes for major cardiovascular events within one year before index date	3509 (8.4%)	850 (15.0%)	1467 (8.0%)	175 (3.5%)	1017 (7.9%)
Diagnosis and procedure codes for bariatric surgery at baseline	139 (0.3%)	26 (0.5%)	36 (0.2%)	51 (1.0%)	26 (0.2%)
Diagnosis codes for congestive heart failure at baseline	2378 (5.7%)	609 (10.8%)	967 (5.3%)	142 (2.8%)	660 (5.1%)
Serum creatinine level >1.4 mg/dL in women and >1.5 mg/dL in men; or kidney transplant codes at baseline; or ≥3 dialysis codes at baseline; or diagnosis code for ESRD at baseline	2073 (5.0%)	429 (7.6%)	919 (5.0%)	98 (2.0%)	627 (4.9%)
Diagnosis codes for cancer (except nonmelanoma skin cancer) at baseline	2570 (6.1%)	464 (8.2%)	1187 (6.5%)	165 (3.3%)	754 (5.9%)
Adherent to oral glucocorticoids for ≥3 months at baseline	599 (1.4%)	145 (2.6%)	252 (1.4%)	42 (0.8%)	160 (1.2%)
A prescription of atypical antipsychotics within 3 months before or on the index date	897 (2.1%)	173 (3.1%)	337 (1.8%)	142 (2.8%)	245 (1.9%)

Table S8. Complete list of medications included in the final cohort.

	Frequency	Percent
Glimepiride	4314	53.92
Liraglutide	690	8.62
Pioglitazone HCL/Glimepiride	3	0.04
Pioglitazone/Glimepiride	1	0.01
Sitagliptin Phos/Metformin HCL	849	10.61
Sitagliptin Phosphate	2144	26.80

Table S9. Baseline characteristics of patients in the four treatment arms prior to weighting.

	Glargine (N=251)	Glimepiride (N=4318)	Liraglutide (N=690)	Sitagliptin (N=2993)	Total (N=8252)	Largest SMD
**Age, mean (SD)**	60.2 (12.4)	62.9 (11.0)	54.9 (9.8)	61.9 (11.2)	61.8 (11.2)	0.67
**Age group, years**						0.81
30-44	30 (12.0%)	268 (6.2%)	97 (14.1%)	237 (7.9%)	632 (7.7%)	
45-54	49 (19.5%)	726 (16.8%)	250 (36.2%)	538 (18.0%)	1563 (18.9%)	
55-64	65 (25.9%)	1125 (26.1%)	221 (32.0%)	783 (26.2%)	2194 (26.6%)	
65-74	81 (32.3%)	1621 (37.5%)	110 (15.9%)	1095 (36.6%)	2907 (35.2%)	
≥75	26 (10.4%)	578 (13.4%)	12 (1.7%)	340 (11.4%)	956 (11.6%)	
**Gender**						0.19
Female	132 (52.6%)	1982 (45.9%)	410 (59.4%)	1499 (50.1%)	4023 (48.8%)	
Male	119 (47.4%)	2336 (54.1%)	280 (40.6%)	1494 (49.9%)	4229 (51.2%)	
**Race/ethnicity**						0.32
White	156 (62.2%)	2834 (65.6%)	483 (70.0%)	1813 (60.6%)	5286 (64.1%)	
Black	39 (15.5%)	555 (12.9%)	100 (14.5%)	381 (12.7%)	1075 (13.0%)	
Hispanic	31 (12.4%)	514 (11.9%)	74 (10.7%)	412 (13.8%)	1031 (12.5%)	
Asian	11 (4.4%)	250 (5.8%)	20 (2.9%)	250 (8.4%)	531 (6.4%)	
Other, unknown, missing	14 (5.6%)	165 (3.8%)	13 (1.9%)	137 (4.6%)	329 (4.0%)	
**Annual household income**						0.46
<$40,000	74 (29.5%)	1090 (25.2%)	125 (18.1%)	663 (22.2%)	1952 (23.7%)	
$40,000 - $74,999	55 (21.9%)	1231 (28.5%)	166 (24.1%)	780 (26.1%)	2232 (27.0%)	
$75,000 – $124,999	73 (29.1%)	1177 (27.3%)	232 (33.6%)	786 (26.3%)	2268 (27.5%)	
$125,000 – $199,999	>11*	437 (10.1%)	>100*	416 (13.9%)	971 (11.8%)	
≥200,000	<11*	159 (3.7%)	>40*	186 (6.2%)	396 (4.8%)	
Unknown/missing	24 (9.6%)	224 (5.2%)	23 (3.3%)	162 (5.4%)	433 (5.2%)	
**Baseline Hb_{A1c}**						0.28
Mean (SD)	7.6 (0.5)	7.7 (0.5)	7.5 (0.5)	7.6 (0.5)	7.6 (0.5)	
Median (IQR)	7.7 (7.2, 8.0)	7.7 (7.3, 8.1)	7.5 (7.1, 7.9)	7.5 (7.2, 8.0)	7.6 (7.2, 8.0)	
**Baseline HbA_{1c} categories**						0.29
6.8-6.9%	27 (10.8%)	295 (6.8%)	100 (14.5%)	289 (9.7%)	711 (8.6%)	
7.0-7.9%	145 (57.8%)	2651 (61.4%)	431 (62.5%)	1943 (64.9%)	5170 (62.7%)	
8.0-8.5%	79 (31.5%)	1372 (31.8%)	159 (23.0%)	761 (25.4%)	2371 (28.7%)	
**Baseline creatinine**						0.25
N (%)	167 (66.5%)	2905 (67.3%)	502 (72.8%)	2248 (75.1%)	5822 (70.6%)	
Mean (SD)	0.9 (0.2)	0.9 (0.2)	0.8 (0.2)	0.9 (0.2)	0.9 (0.2)	

Median (IQR)	0.9 (0.7, 1.0)	0.9 (0.7, 1.0)	0.8 (0.7, 1.0)	0.9 (0.7, 1.0)	0.9 (0.7, 1.0)	
**Baseline comorbidities**						
Nephropathy	41 (16.3%)	412 (9.5%)	41 (5.9%)	243 (8.1%)	737 (8.9%)	0.33
Retinopathy	22 (8.8%)	205 (4.7%)	29 (4.2%)	166 (5.5%)	422 (5.1%)	0.19
Neuropathy	55 (21.9%)	561 (13.0%)	84 (12.2%)	325 (10.9%)	1025 (12.4%)	0.30
Hyperglycemia	<11*	<11*	0 (0.0%)	0 (0.0%)	<11*	0.18
Hypoglycemia	<11*	<11*	0 (0.0%)	0 (0.0%)	<11*	0.09
Coronary artery disease	26 (10.4%)	395 (9.1%)	38 (5.5%)	247 (8.3%)	706 (8.6%)	0.18
Chronic kidney disease	21 (8.4%)	141 (3.3%)	11 (1.6%)	92 (3.1%)	265 (3.2%)	0.32
Cerebrovascular disease	<11*	126 (2.9%)	≥11*	97 (3.2%)	246 (3.0%)	0.07
Peripheral vascular disease	21 (8.4%)	217 (5.0%)	21 (3.0%)	141 (4.7%)	400 (4.8%)	0.23
**Baseline medications**						
Statin	145 (57.8%)	2976 (68.9%)	415 (60.1%)	2092 (69.9%)	5628 (68.2%)	0.25
Non-statin lipid lower medications	31 (12.4%)	516 (11.9%)	99 (14.3%)	464 (15.5%)	1110 (13.5%)	0.09
ACE inhibitor	119 (47.4%)	1958 (45.3%)	279 (40.4%)	1225 (40.9%)	3581 (43.4%)	0.14
Angiotensin receptor blocker	44 (17.5%)	1121 (26.0%)	201 (29.1%)	829 (27.7%)	2195 (26.6%)	0.28
ACE inhibitor or angiotensin receptor blocker	157 (62.5%)	2994 (69.3%)	465 (67.4%)	1997 (66.7%)	5613 (68.0%)	0.14
Sacubitril /Valsartan	0 (0.0%)	0 (0.0%)	0 (0.0%)	0 (0.0%)	0 (0.0%)	--
Warfarin	<11*	93 (2.2%)	≥11*	45 (1.5%)	160 (1.9%)	0.06
Direct oral anti-coagulant	<11*	56 (1.3%)	≥11*	51 (1.7%)	128 (1.6%)	0.08
Peripheral neuropathy medications	46 (18.3%)	421 (9.7%)	79 (11.4%)	304 (10.2%)	850 (10.3%)	0.25
**Specialty of treating physicians**						0.45
Primary care	153 (61.0%)	3429 (79.4%)	450 (65.2%)	2204 (73.6%)	6236 (75.6%)	
Endocrinology	28 (11.2%)	148 (3.4%)	75 (10.9%)	140 (4.7%)	391 (4.7%)	
Cardiology	<11*	>20*	<11*	≥20*	58 (0.7%)	
Nephrology	<11*	<11*	<11*	<11*	15 (0.2%)	
Other	20 (8.0%)	246 (5.7%)	102 (14.8%)	192 (6.4%)	560 (6.8%)	
Unknown	49 (19.5%)	460 (10.7%)	59 (8.6%)	424 (14.2%)	992 (12.0%)	
**Year of cohort entry**						0.27
2010	<11*	236 (5.5%)	≥30*	191 (6.4%)	477 (5.8%)	
2011	14 (5.6%)	216 (5.0%)	37 (5.4%)	224 (7.5%)	491 (6.0%)	
2012	19 (7.6%)	275 (6.4%)	40 (5.8%)	253 (8.5%)	587 (7.1%)	
2013	23 (9.2%)	357 (8.3%)	73 (10.6%)	319 (10.7%)	772 (9.4%)	

2014	21 (8.4%)	423 (9.8%)	71 (10.3%)	397 (13.3%)	912 (11.1%)	
2015	22 (8.8%)	514 (11.9%)	75 (10.9%)	252 (8.4%)	863 (10.5%)	
2016	41 (16.3%)	635 (14.7%)	116 (16.8%)	343 (11.5%)	1135 (13.8%)	
2017	42 (16.7%)	861 (19.9%)	119 (17.2%)	471 (15.7%)	1493 (18.1%)	
2018	49 (19.5%)	670 (15.5%)	96 (13.9%)	446 (14.9%)	1261 (15.3%)	
2019	<11*	131 (3.0%)	≥20*	97 (3.2%)	261 (3.2%)	

Abbreviations: SMD, standardized mean difference
* Cell suppression based on OptumLabs Cell Size Suppression Rules. N<11 are masked to protect patient confidentiality

Table S10. Pairwise standardized mean difference for weighted cohort.

	Glimepiride vs Liraglutide	Glimepiride vs Sitagliptin	Liraglutide vs Sitagliptin
**Age**	0.14	0.00	0.14
**Sex**	0.05	0.02	0.03
**Race/ethnicity**	0.08	0.02	0.09
**Annual Household Income**	0.10	0.03	0.10
**Baseline HbA_{1c}**	0.06	0.03	0.03
**Baseline HbA_{1c} categories**	0.09	0.02	0.07
**Baseline creatinine**	0.02	0.00	0.02
**Baseline comorbidities**			
Nephropathy	0.03	0.02	0.05
Retinopathy	0.03	0.01	0.03
Neuropathy	0.03	0.01	0.05
Hyperglycemia	0.03	0.03	0.00
Hypoglycemia	0.03	0.03	0.00
Coronary artery disease	0.02	0.02	0.04
Chronic kidney disease	0.01	0.00	0.01
Cerebrovascular disease	0.00	0.00	0.00
Peripheral vascular disease	0.02	0.00	0.01
**Baseline medications**			
ACE inhibitor	0.00	0.01	0.01
ARB	0.02	0.01	0.01
ACE inhibitor or ARB	0.03	0.01	0.04
Direct oral anticoagulant	0.01	0.00	0.01
Statin	0.07	0.02	0.08
Non-statin lipid lowering medications	0.05	0.01	0.04
Warfarin	0.00	0.02	0.02
Peripheral neuropathy medications	0.09	0.01	0.09
**Specialty of treating physicians**	0.10	0.02	0.10
**Year of cohort entry**	0.13	0.03	0.13

Figure S4. Inverse probability of treatment weighted Kaplan Meier curve for the time to right censoring by treatment groups.

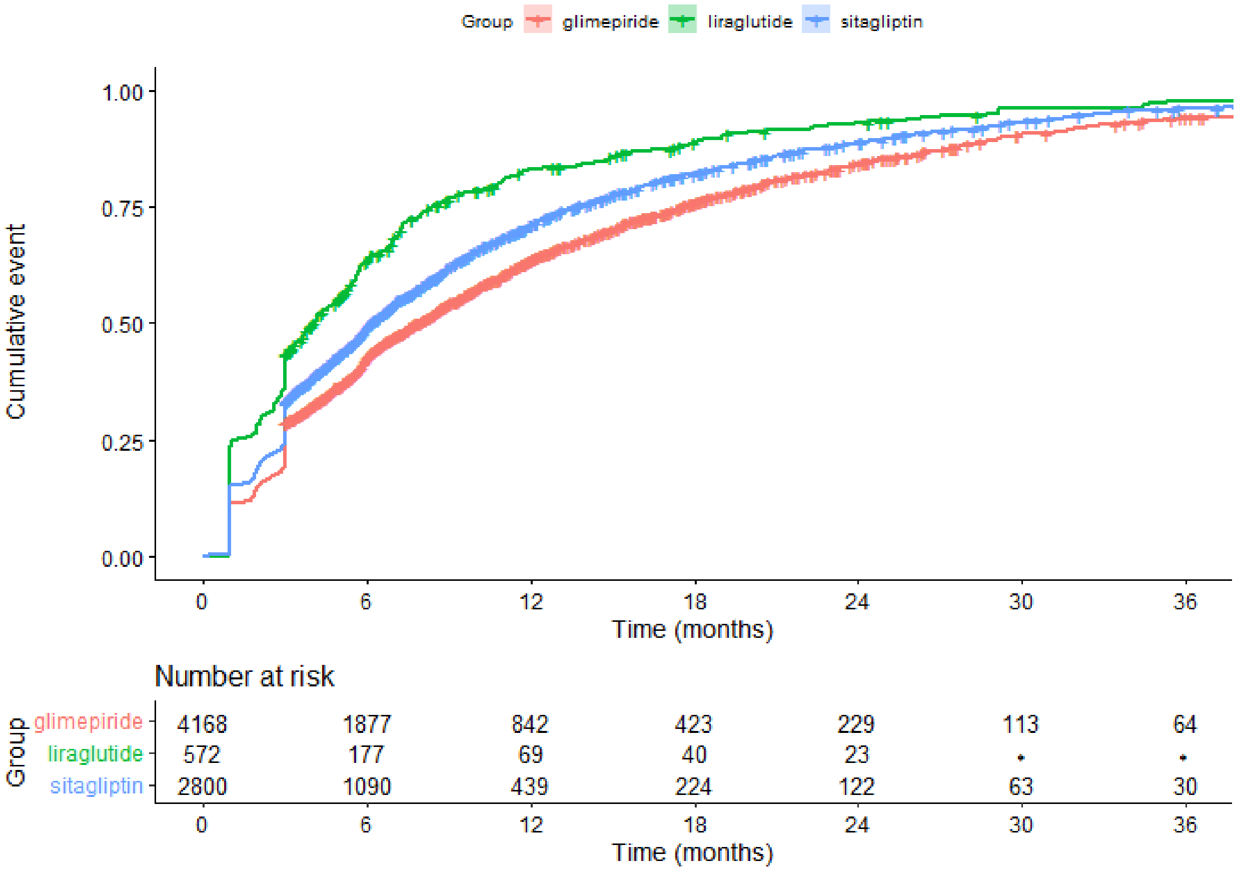

Figure S5. Cumulative risks of secondary metabolic failure in propensity score weighted patients. Censor per-protocol primary analysis.

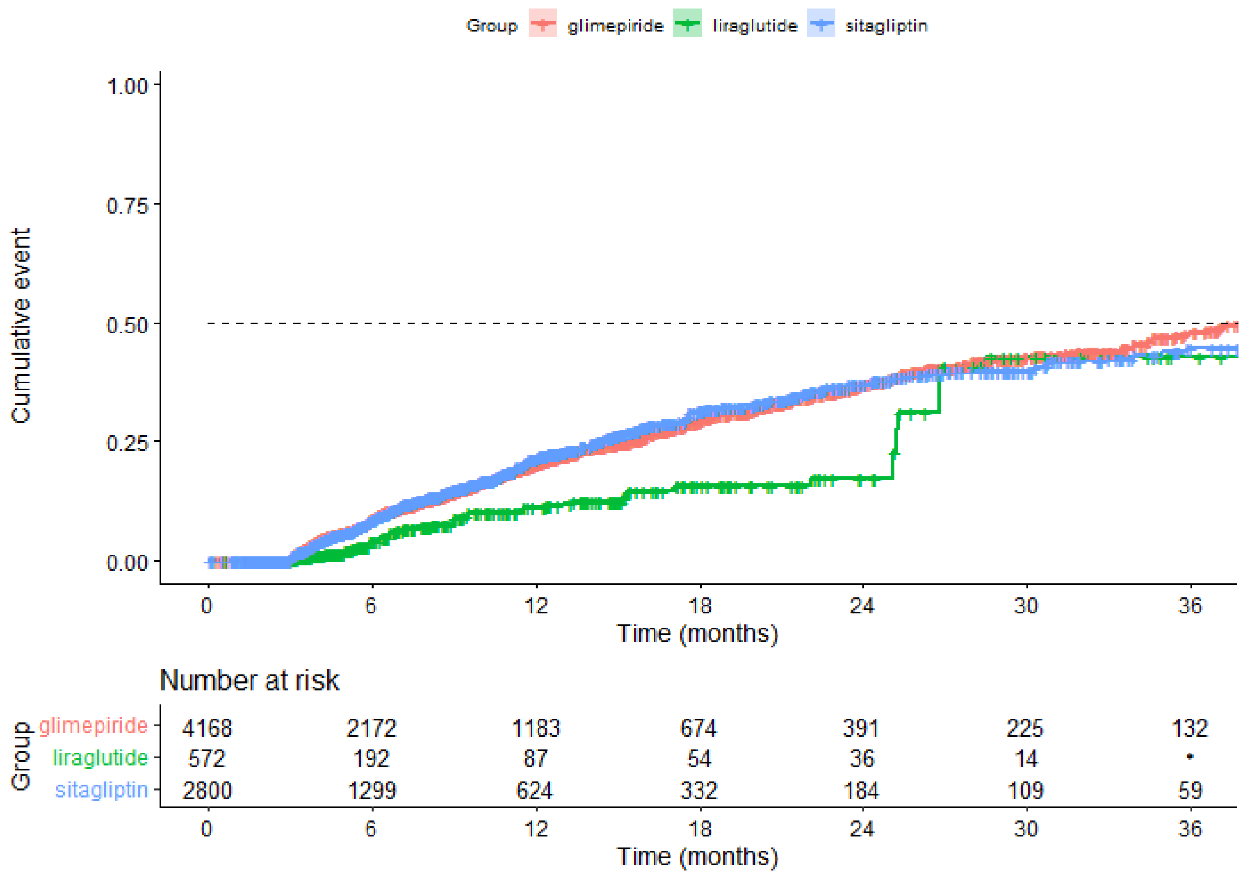

Time to secondary metabolic failure	First Quintile	Median
Glimepiride	472 (95% CI, 415-498)	1237 (95% CI, 1054-1478)
Liraglutide	768 (95% CI, 764-NA)	1601 (95% CI, 815-NA)
Sitagliptin	437 (95% CI, 383-483)	1521 (95% CI, 1155-NA)

Table S11. Rates of secondary microvascular, macrovascular, and safety endpoints (weighted). Rates of end-stage kidney disease, heart failure, pancreatitis, pancreatic/thyroid cancer, and all-cause mortality are not shown because there were fewer than 11 crude (unweighted) events in at least one of the treatment arms.

	No. events	Event rate per 100 person-years
**Retinopathy**		
Glimepiride	188	1.83
Liraglutide	31	2.16
Sitagliptin	119	1.70
Total	338	1.80
**Neuropathy**		
Glimepiride	609	5.92
Liraglutide	97	6.76
Sitagliptin	364	5.19
Total	1070	5.70
**MACE**		
Glimepiride	158	1.54
Liraglutide	25	1.74
Sitagliptin	96	1.37
Total	279	1.49
**Other Cardiovascular events**		
Glimepiride	204	1.98
Liraglutide	33	2.30
Sitagliptin	122	1.74
Total	359	1.91
**Cancer**		
Glimepiride	175	1.70
Liraglutide	27	1.88
Sitagliptin	124	1.77
Total	326	1.74
**Any hospital admission**		
Glimepiride	734	7.13
Liraglutide	102	7.11
Sitagliptin	480	6.84
Total	1316	7.02

Table S12. Hazard ratios of secondary microvascular, macrovascular, and safety endpoints.

	HR (95% CI)	Holm adjusted p-value
**Retinopathy**		
Liraglutide vs Glimepiride	1.22 (0.75 to 1.96)	0.84
Sitagliptin vs Glimepiride	0.93 (0.73 to 1.17)	0.84
Liraglutide vs Sitagliptin	1.31 (0.80 to 2.14)	0.83
**Neuropathy**		
Liraglutide vs Glimepiride	1.18 (0.90 to 1.55)	0.23
Sitagliptin vs Glimepiride	0.87 (0.76 to 0.99)	0.09
Liraglutide vs Sitagliptin	1.36 (1.03 to 1.80)	0.09
**MACE**		
Liraglutide vs Glimepiride	1.13 (0.61 to 2.10)	1.00
Sitagliptin vs Glimepiride	0.89 (0.69 to 1.15)	1.00
Liraglutide vs Sitagliptin	1.27 (0.67 to 2.40)	1.00
**Other Cardiovascular events**		
Liraglutide vs Glimepiride	1.17 (0.71 to 1.94)	0.70
Sitagliptin vs Glimepiride	0.87 (0.69 to 1.09)	0.70
Liraglutide vs Sitagliptin	1.35 (0.80 to 2.27)	0.70
**Cancer**		
Liraglutide vs Glimepiride	1.11 (0.65 to 1.89)	1.00
Sitagliptin vs Glimepiride	1.04 (0.83 to 1.31)	1.00
Liraglutide vs Sitagliptin	1.06 (0.62 to 1.83)	1.00
**Any hospital admission**		
Liraglutide vs Glimepiride	1.02 (0.78 to 1.33)	1.00
Sitagliptin vs Glimepiride	0.95 (0.85 to 1.07)	1.00
Liraglutide vs Sitagliptin	1.07 (0.81 to 1.40)	1.00

Table S13. Subgroup Analyses for Primary Metabolic Failure.

	Hazard ratio (95% CI)	Holm adjusted p-value
**Baseline HbA_{1c} level**		
**Baseline HbA_{1c} < 7.0%**		
Liraglutide vs Glimepiride	0.21 (0.06 to 0.79)	0.06
Sitagliptin vs Glimepiride	0.93 (0.61 to 1.43)	0.75
Liraglutide vs Sitagliptin	0.23 (0.06 to 0.85)	0.06
**Baseline HbA_{1c} ≥ 7.0%**		
Liraglutide vs Glimepiride	0.59 (0.44 to 0.78)	<0.001
Sitagliptin vs Glimepiride	1.04 (0.94 to 1.14)	0.44
Liraglutide vs Sitagliptin	0.58 (0.43 to 0.79)	<0.001
**Age group**		
**Age < 65**		
Liraglutide vs Glimepiride	0.54 (0.42 to 0.71)	<0.001
Sitagliptin vs Glimepiride	0.93 (0.82 to 1.07)	0.30
Liraglutide vs Sitagliptin	0.58 (0.44 to 0.77)	<0.001
**Age ≥ 65**		
Liraglutide vs Glimepiride	0.57 (0.31 to 1.06)	0.10
Sitagliptin vs Glimepiride	1.14 (1.00 to 1.29)	0.10
Liraglutide vs Sitagliptin	0.50 (0.27 to 0.94)	0.09
**Gender**		
**Female**		
Liraglutide vs Glimepiride	0.49 (0.33 to 0.74)	0.002
Sitagliptin vs Glimepiride	0.99 (0.87 to 1.13)	0.88
Liraglutide vs Sitagliptin	0.50 (0.33 to 0.75)	0.002
**Male**		
Liraglutide vs Glimepiride	0.63 (0.42 to 0.93)	0.04
Sitagliptin vs Glimepiride	1.07 (0.94 to 1.22)	0.31
Liraglutide vs Sitagliptin	0.59 (0.39 to 0.88)	0.03
**Race/Ethnicity**		
**White**		
Liraglutide vs Glimepiride	0.58 (0.42 to 0.81)	0.002
Sitagliptin vs Glimepiride	1.06 (0.94 to 1.19)	0.34
Liraglutide vs Sitagliptin	0.55 (0.40 to 0.77)	0.001
**Black**		
Liraglutide vs Glimepiride	0.57 (0.23 to 1.41)	0.65
Sitagliptin vs Glimepiride	0.84 (0.64 to 1.11)	0.65
Liraglutide vs Sitagliptin	0.68 (0.27 to 1.70)	0.65
**Hispanic**		
Liraglutide vs Glimepiride	0.10 (0.03 to 0.35)	<0.001
Sitagliptin vs Glimepiride	1.24 (0.95 to 1.60)	0.11

Liraglutide vs Sitagliptin	0.08 (0.03 to 0.29)	<0.001
**Asian**		
Liraglutide vs Glimepiride	0.84 (0.35 to 2.03)	1.00
Sitagliptin vs Glimepiride	0.70 (0.48 to 1.01)	0.17
Liraglutide vs Sitagliptin	1.20 (0.49 to 2.94)	1.00

Table S14. Subgroup Analyses for Secondary Metabolic Failure.

	HR (95% CI)	Holm adjusted p-value
**Baseline HbA_{1c} level**		
**Baseline HbA_{1c} < 7.0%**		
Liraglutide vs Glimepiride	0.28 (0.05 to 1.55)	0.43
Sitagliptin vs Glimepiride	0.81 (0.43 to 1.54)	0.53
Liraglutide vs Sitagliptin	0.34 (0.06 to 1.92)	0.45
**Baseline HbA_{1c} ≥ 7.0%**		
Liraglutide vs Glimepiride	0.62 (0.43 to 0.90)	0.02
Sitagliptin vs Glimepiride	1.04 (0.91 to 1.19)	0.53
Liraglutide vs Sitagliptin	0.60 (0.41 to 0.87)	0.02
**Age group**		
**Age < 65**		
Liraglutide vs Glimepiride	0.53 (0.36 to 0.78)	0.004
Sitagliptin vs Glimepiride	0.98 (0.81 to 1.18)	0.80
Liraglutide vs Sitagliptin	0.54 (0.36 to 0.81)	0.005
**Age ≥ 65**		
Liraglutide vs Glimepiride	0.68 (0.34 to 1.38)	0.62
Sitagliptin vs Glimepiride	1.08 (0.90 to 1.30)	0.62
Liraglutide vs Sitagliptin	0.63 (0.31 to 1.29)	0.62
**Gender**		
**Female**		
Liraglutide vs Glimepiride	0.57 (0.35 to 0.91)	0.06
Sitagliptin vs Glimepiride	0.99 (0.82 to 1.20)	0.94
Liraglutide vs Sitagliptin	0.57 (0.35 to 0.93)	0.06
**Male**		
Liraglutide vs Glimepiride	0.65 (0.38 to 1.12)	0.24
Sitagliptin vs Glimepiride	1.08 (0.90 to 1.29)	0.41
Liraglutide vs Sitagliptin	0.60 (0.39 to 0.91)	0.22
**Race/Ethnicity**		
**White**		
Liraglutide vs Glimepiride	0.64 (0.42 to 0.97)	0.07
Sitagliptin vs Glimepiride	1.08 (0.92 to 1.27)	0.35
Liraglutide vs Sitagliptin	0.61 (0.39 to 0.95)	0.05
**Black**		
Liraglutide vs Glimepiride	0.40 (0.15 to 1.09)	0.22
Sitagliptin vs Glimepiride	0.86 (0.59 to 1.25)	0.42
Liraglutide vs Sitagliptin	0.47 (0.17 to 1.30)	0.29
**Hispanic**		
Liraglutide vs Glimepiride	0.13 (0.03 to 0.63)	0.03
Sitagliptin vs Glimepiride	0.97 (0.67 to 1.41)	0.88
Liraglutide vs Sitagliptin	0.14 (0.03 to 0.65)	0.03

**Asian**		
Liraglutide vs Glimepiride	1.71 (0.89 to 3.29)	0.21
Sitagliptin vs Glimepiride	0.83 (0.47 to 1.44)	0.50
Liraglutide vs Sitagliptin	2.08 (1.05 to 4.10)	0.10

Figure S6. Cumulative risks of primary and secondary metabolic failure (sensitivity analysis). Sensitivity analysis conducted with as-treated censoring.

Primary metabolic failure

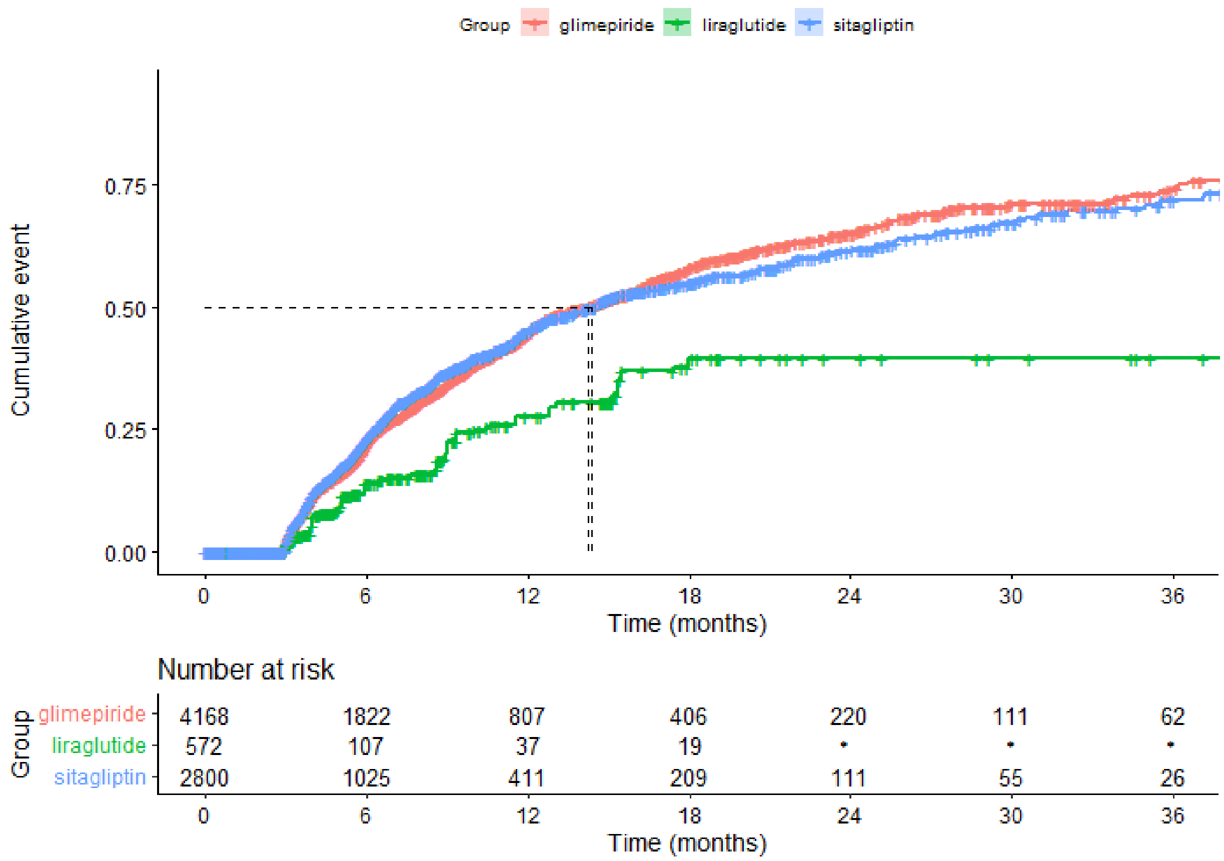

Secondary metabolic failure

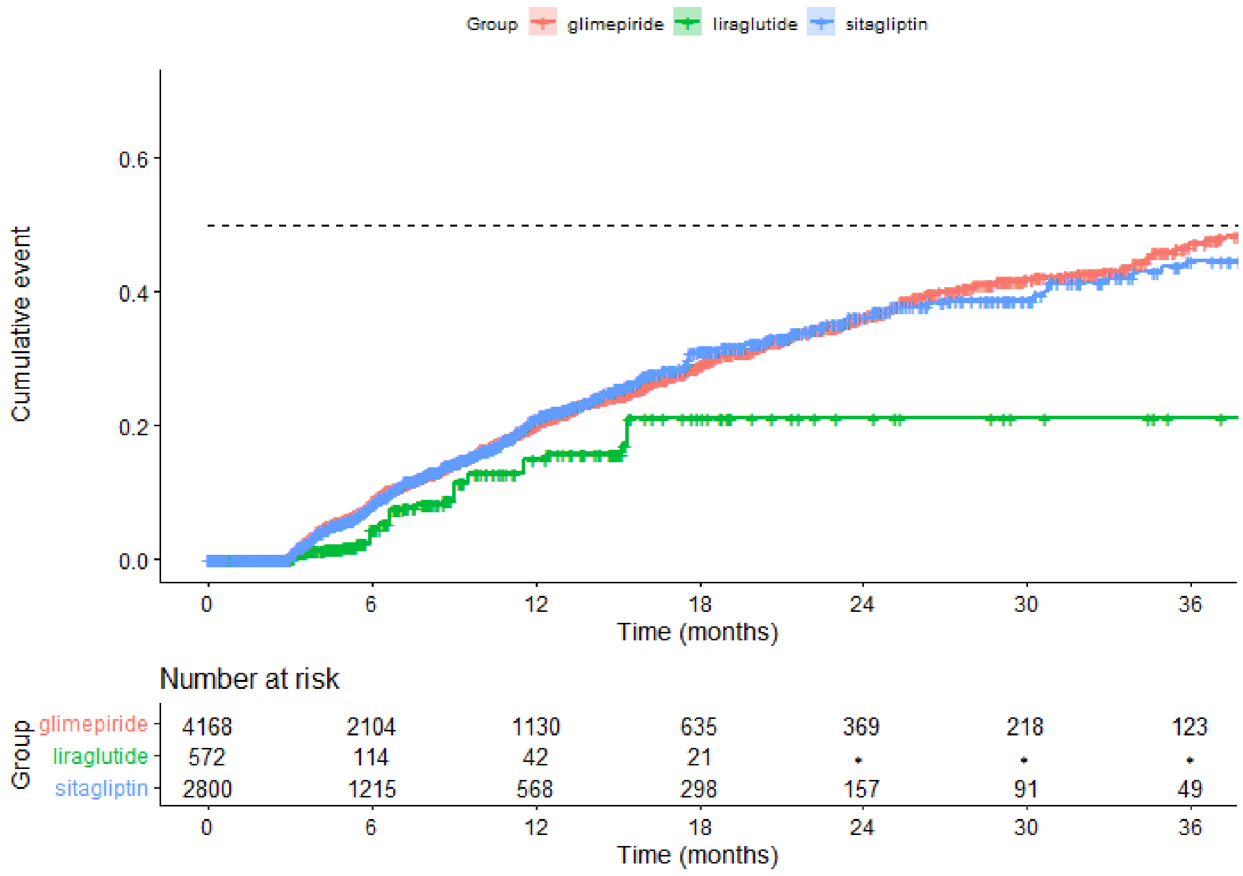

Table S15. Hazard ratios for primary and secondary metabolic failure (sensitivity analysis). Sensitivity analysis conducted with as-treated censoring.

	HR (95% CI)	Holm adjusted p-value
**Primary metabolic failure**		
Liraglutide vs Glimepiride	0.43 (0.35 to 0.80)	0.006
Sitagliptin vs Glimepiride	1.03 (0.94 to 1.13)	0.56
Liraglutide vs Sitagliptin	0.51 (0.34 to 0.78)	0.006
**Secondary metabolic failure**		
Liraglutide vs Glimepiride	0.59 (0.33 to 1.07)	0.20
Sitagliptin vs Glimepiride	1.03 (0.90 to 1.19)	0.62
Liraglutide vs Sitagliptin	0.57 (0.31 to 1.04)	0.20
**Initiating insulin**		
Liraglutide vs Glimepiride	0.57 (0.20 to 1.64)	0.59
Sitagliptin vs Glimepiride	1.17 (0.77 to 1.78)	0.59
Liraglutide vs Sitagliptin	0.49 (0.17 to 1.42)	0.56

Table S16. Falsification end point analysis. Falsification endpoint was any office visit, emergency department, or hospital claim for pneumonia during the follow-up period.

	HR (95% CI)	Holm adjusted p-value
Liraglutide vs Glimepiride	1.01 (0.59 to 1.73)	1.00
Sitagliptin vs Glimepiride	0.97 (0.79 to 1.21)	1.00
Liraglutide vs Sitagliptin	1.04 (0.61 to 1.80)	1.00

Rationale: Our objective in choosing a falsification endpoint was to identify an outcome that is 1) measurable in our data, 2) is not related to any of the study outcomes, including glycemic control, microvascular complications, and macrovascular complications; and 3) is not related to glucose-lowering medications. Pneumonia meets each of these criteria. It also has a similar bias structure as HbA_{1c}, as more clinically complex and/or sicker patients may be more likely to experience pneumonia and also to experience deterioration in glycemic control, have their HbA_{1c} tested more frequently, and experience microvascular and macrovascular complications.

REFERENCES

1. McCaffrey DF, Griffin BA, Almirall D, et al. A tutorial on propensity score estimation for multiple treatments using generalized boosted models. *Statistics in medicine* 2013;32(19):3388-414. doi: 10.1002/sim.5753 [published Online First: 2013/03/18]
2. Greenwell B, Boehmke B, Cunningham J, et al. gbm: Generalized Boosted Regression Models: R package version 2.1.8, 2020.
3. American Diabetes Association Standards of Medical Care in Diabetes—2022. Section 6. Glycemic Targets. *Diabetes Care* 2021;45(Supplement_1):S83-S96. doi: 10.2337/dc22-S006
